# Discovery of Novel Entomopathogenic Fungi for Mosquito-Borne Disease Control

**DOI:** 10.3389/ffunb.2021.637234

**Published:** 2021-07-27

**Authors:** Anastasia Accoti, Cecilia Springer Engdahl, George Dimopoulos

**Affiliations:** W. Harry Feinstone Department of Molecular Microbiology and Immunology, Bloomberg School of Public Health, Johns Hopkins University, Baltimore, MD, United States

**Keywords:** *Anopheles gambiae*, *Aedes aegypti*, entomopathogenic fungi, biopesticides, mosquito control, vector-borne diseases

## Abstract

The increased application of chemical control programs has led to the emergence and spread of insecticide resistance in mosquitoes. Novel environmentally safe control strategies are currently needed for the control of disease vectors. The use of entomopathogenic fungi could be a suitable alternative to chemical insecticides. Currently, *Beauveria* spp. and *Metarhizium* spp. are the most widely used entomopathogenic fungi for mosquito control, but increasing the arsenal with additional fungi is necessary to mitigate the emergence of resistance. Entomopathogenic fungi are distributed in a wide range of habitats. We have performed a comprehensive screen for candidate mosquitocidal fungi from diverse outdoor environments in Maryland and Puerto Rico. An initial screening of 22 fungi involving exposure of adult *Anopheles gambiae* to 2-weeks-old fungal cultures identified five potent pathogenic fungi, one of which is unidentified and the remaining four belonging to the three genera *Galactomyces* sp., *Isaria* sp. and *Mucor* sp. These fungi were then screened against *Aedes aegypti*, revealing *Isaria* sp. as a potent mosquito killer. The entomopathogenic effects were confirmed through spore-dipping assays. We also probed further into the killing mechanisms of these fungi and investigated whether the mosquitocidal activities were the result of potential toxic fungus-produced metabolites. Preliminary assays involving the exposure of mosquitoes to sterile filtered fungal liquid cultures showed that *Galactomyces* sp., *Isaria* sp. and the unidentified isolate 1 were the strongest producers of factors showing lethality against *An. gambiae*. We have identified five fungi that was pathogenic for *An. gambiae* and one for *Ae. aegypti*, among these fungi, four of them (two strains of *Galactomyces* sp., *Mucor sp.*, and the unidentified isolate 1) have never previously been described as lethal to insects. Further characterization of these entomopathogenic fungi and their metabolites needs to be done to confirm their potential use in biologic control against mosquitoes.

## Introduction

Vector-borne disease accounts for more than 17% of all infectious disease, causing more than 700,000 deaths annually. *Anopheles gambiae* is the most important malaria vector, and *Aedes aegypti* is responsible for the transmission of dengue fever, Zika virus disease, chikungunya, Rift Valley fever, and yellow fever, among others [World Health Organization (WHO), 2020]. Current mosquito vector control strategies are primarily based on synthetic insecticides. However, the increased implementation of chemical control programs has led to the emergence and spread of insecticide resistance in *Anopheles* sp. *(An.)* and *Aedes* sp. *(Ae.)* populations and is also affecting non-target organisms, including humans (Moyes et al., 2017; Riveron et al., 2018).

Several studies have demonstrated the potential of using entomopathogenic fungi for controlling mosquito vectors (Charnley and Collins, 2007), as an effective and environmentally safe strategy. Since one of the mode of action of entomopathogenic fungi is mediated through surface contact with adult mosquitoes, these agents would be applicable to a variety of deployment strategies, some of which are already in use for chemical insecticides (Scholte et al., 2005; Farenhorst et al., 2011). More importantly, they are effective against mosquito strains that have developed resistance to the available chemical insecticides (Farenhorst et al., 2009, 2010; Blanford et al., 2011). Furthermore, in contrast to chemical insecticides that generally kill mosquitoes within 24 h, fungal biopesticides usually require more than a week to kill exposed mosquitoes and thereby the probability is decreased that resistance will emerge (Ffrench-Constant, 2005; Read et al., 2009).

*Metarhizium* spp. and *Beauveria* spp. are the best characterized and most widely used fungi in biological control programs (Scholte et al., 2005; Hancock, 2009; Knols et al., 2010; Blanford et al., 2011; Farenhorst et al., 2011). About 13 species and sub-species of both these fungi have been formulated and registered as mycoinsecticides or mycoacaricides (de Faria and Wraight, 2007). While their mode of action against adult mosquitoes is based on slow and gradual penetration and invasion through the invertebrate cuticle, the emergence of resistance is still a likely event, as has been demonstrated by Dubovskiy et al. (2013). It is therefore necessary to expand the repertoire of fungal biopesticides with additional fungi in order to mitigate the emergence of resistance; the availability of additional fungal biopesticides will potentially also enable a greater versatility of exposure routes that may be dependent of the biology of the fungus. Key stages of the infection process by entomopathogenic fungi are: (a) adhesion of infectious spores to the surface of the insect cuticle; (b) penetration of the cuticle via enzymes and mechanical force; (c) colonization of the hemocoel; and (d) emergence of the conidiophores for external sporulation on the insect cadaver (Butt et al., 2016). In addition, entomopathogenic fungi produce a variety of secondary metabolites, some of which are highly toxic to the larval, pupal, and adult mosquito stages, as well as to some pathogens such as the malaria parasite (Singh and Prakash, 2012; Niu et al., 2015; Vivekanandhan et al., 2018a, 2020a). A better understanding of the toxic metabolites produced by fungi can help unravel the relevant mosquitocidal mechanisms and enable the development of novel natural product-based mosquito control agents.

Entomopathogenic fungi are present in a wide range of habitats (Lacey and Fransen, 1996; Chandler et al., 1997; Sánchez-Peña et al., 2011). Here we aimed at discovering novel mosquitocidal fungi from diverse habitats with activity against *An. gambiae* and *Ae. aegypti* adults. We use three different ways to expose adult mosquitoes to the isolated environmental fungi, the first exposure assay was primarily used as a screening method to differentiate pathogenic from non-pathogenic environmental fungi. This method was a direct exposure of adult mosquitoes to fungal cultures on agar plates containing fungal mycelia, the growing hyphae, spores and possible toxic compounds all together. The second methodology involved the use of purified spores in solution (10^8^ spores/ml) alone. The third way to expose mosquitoes, to determine whether the killing effect is mediated by toxic metabolites and/or requires actual exposure to live fungi, was sugar fed adult mosquitoes with a sugar solution containing only the fungal metabolites.

## Results

### Fungus Sample Collection

To maximize the probability of identifying novel fungi with entomopathogenic properties, samples of fungus-containing plants, decomposing material, soil, and stagnant water were collected from outdoor mosquito habitats in Maryland (MD) and Puerto Rico (Table 1).

**Table 1:** Collection and identification of fungi.

**Ref Nr^a^**	**ITS-based identification**				**Collection**		**Entomopathogenic activity**	
	**Fungi**	**Query cover(%)**	**Query identity (%)**	**Accesionnumber**	**Country (MD/PR)^b^**	**Source**	**Known EPF^c^ (Y/N)^d^**	**EPF^c^ againstmosquitoes(Y/N)^d^**
1	*Isolate* 1 sp.	99	98,9	*MT786363.1*	MD	Water pond	**N**	**N**
2	Ascomycota	95	99,8	KT315401.1	PR	Leaf	**Y** ^1,2,3,4,5,6^	
3	*Aureobasidium* sp.	88	100	MK156692.1	PR	Leaf	**N**	**N**
4	*Cercospora* sp.	100	99,8	AY633838.1	PR	Leaf	**N**	**N**
5	*Cladosporium* sp.	100	99,6	MT508793.1	PR	Leaf	**Y** ^7^	**N**
6	*Cladosporium* sp.	100	100	MT582794.1	MD	Container	**Y** ^8^	**N**
7	*Colletotrichum* sp.	100	99,8	MN889467.1	PR	Leaf	**N**	**N**
8	*Fusarium* 1 sp.	100	99,6	MT598827.1	PR	Leaf	**Y** ^9,10^	**Y** ^11^
9	*Fusarium* 2 sp.	99	100	MW553789.1	PR	Moss		
10	*Fusarium* 3 sp.	100	99,6	MT603301.1	PR	Flower		
11	*Fusarium* 4 sp.	100	99,8	MW582382.1	MD	Fountain		
12	*Galactomyces* 1 sp.	99	100	MF044044.1	MD	Soil	**N**	**N**
13	*Galactomyces* 2 sp.	100	99,7	DQ907937.1	MD	Soil		
14	*Isaria* sp.	100	100	KX241857.1	MD	Container	**Y** ^12,13,14,15,16^	**Y** ^16,17,18,19,20,21^
15	*Mucor* 1 sp.	100	99,6	MN087659.1	PR	Dec Material^e^	**N**	**N**
16	*Mucor* 2 sp.	100	99,6	MN905930.1	MD	Container	**N**	**N**
17	*Mucor* 3 sp.	100	100	MK594384.1	MD	Fountain	**Y** ^22^	**N**
18	*Penicillium* sp.	100	99,4	AF125944.1	PR	Leaf	**Y** ^23^	
19	*Phomopsis* 1 sp.	100	100	MT071116.1	PR	Leaf	**Y** ^24,25^	**N**
20	*Phomopsis* 2 sp.	100	99,6	MT071116.1	PR	Leaf		
21	*Pleosporales* sp.	100	100	JN851029.1	PR	Leaf	**N**	**N**
22	*Scopulariopsis* sp.	99	100	MT609891.1	PR	Dec Material^e^	**Y** ^26^	**N**

a *Ref Nr, Reference Number*;

b *Locality, Samples were collected in Maryland (MD) or in Puerto Rico (PR)*.

c *EPF, entomopathogenic fungi*;

d *Y/N, Yes or No*;

e *Dec Material, Decomposing Material*;

1 *Farenhorst et al. (2011)*;

2 *Hancock (2009)*;

3 *Hancock et al. (2009)*;

4 *Knols et al. (2010)*;

5 *Blanford et al. (2005)*;

6 *Scholte et al. (2005)*,

7 *Singh et al. (2015)*;

8 *Elbanhawy et al. (2019)*;

9 *Chehri (2017)*;

10 *Pelizza et al. (2011)*;

11 *Vivekanandhan et al. (2018a)*;

12 *United States Environmental Protection Agency (EPA). (2020)*;

13 *European Commission (EU) (2020)*;

14 *Ministerio de Agricultura (2020)*;

15 *Zhang et al. (2016)*;

16 *Xu et al. (2017)*;

17 *Luz et al. (2007)*;

18 *Leles et al. (2010)*;

19 *Blanford et al. (2012)*;

20 *Ramirez et al. (2018)*;

21 *Banu and Balasubramanian (2014)*;

22 *Konstantopoulou et al. (2006)*;

23 *Maketon et al. (2014)*;

24 *Meepagala et al. (2018)*;

25 *Amatuzzi et al. (2018)*;

26 *Niu et al. (2019)*.

In Baltimore, MD, samples were collected from six different sources, including stagnant water from containers, a fountain, and a pond, and from soil in an outdoor pot and two tree flowers. In the rural area of Maunabo, Puerto Rico, 30 fungal samples were collected from 15 different types of leaves, five different decomposing materials, nine types of flowers, and one sample was collected from moss.

### Culturing and Identification of Fungi

**Figure 1:**
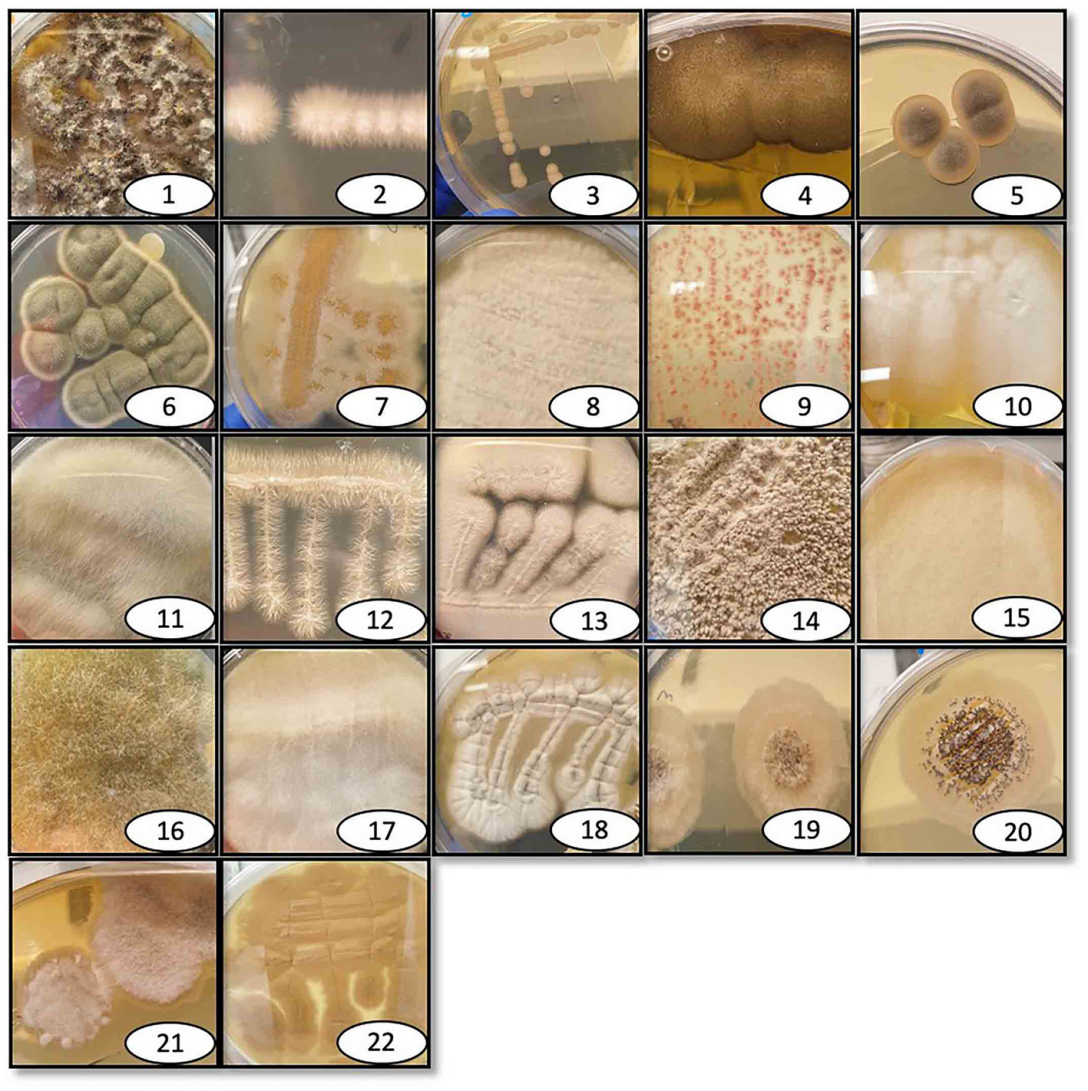
Pictures of isolated and identified fungi. Individual photographs of the isolated fungi that are identified here, labeled with the reference number, and listed in Table 1. The fungi were collected at various outdoor locations and maintained in the lab on either rich growth agar medium brain heart infusion or Sabouraud dextrose, depending on the fungal species.

The 36 environmental samples were manually homogenized and plated on rich fungal solid growth medium to allow the growth of as many fungi as possible. Each morphologically distinct fungal colony was re-streaked on a new agar plate until an axenic fungal culture was attained for each of the different fungal colonies. Once these were separated and streaked on individual plates, several isolates did not grow for more than one generation and were thus excluded from the study. Possible explanations for this could be that these isolates had an obligate symbiosis relationship with microorganisms in the collected sample, or that they could not produce a sufficient number of spores necessary to propagate under laboratory conditions. The 36 collected samples generated a total of 76 fungal isolates, 22 of which could be successfully cultured under the specified laboratory conditions (Figure 1). DNA was extracted from these 22 fungi and used for ribosomal 18S gene amplification and sequencing for identification (Table 1). After the DNA extraction, ribosomal 18S gene amplification was performed using internal transcribed spacer (ITS) degenerate primers, with either of two forward primers (ITS1, ITS3) in combination with one common reverse primer (ITS4) (Supplementary Table 1).

The sequence identity was determined through BLAST nucleotide searches against the NCBI database (Table 1). The identity of the fungi was determined through analysing phylogenetic trees constructed using MEGAX software with the the Bootstrap and the Neighbor-joining statistic method (Supplementary Files 1–3).

As many as 20 of the 22 isolated fungi were identified down to genus level using a cut-off of 98% for query coverage and identity. One isolate (Ascomycota) was identified only at the phylum level, and the identity of another isolate was ambiguous and was therefore called fungal isolate 1. The top hit for fungal isolate 1 when BLASTed was *Aaosphaeria* sp., but as can be seen in the phylogenetic tree this result was not conclusive as several other genera appears equally identical. The 22 fungal isolates belonged to the following 12 different genera and one phylum: phylum Ascomycota*, Aureobasidium* sp., *Cercospora* sp., *Cladosporium* sp., *Colletotrichum* sp., *Fusarium* sp., *Galactomyces* sp., *Isaria* sp., *Mucor* sp., *Penicillium* sp., *Phomopsis* sp., *Pleosporales* sp. and *Scopulariopsis* sp. (ITS-based sequences are presented in Supplementary File 1). Some of the identified fungi have different nomenclature depending on the sexual stage; *Galactomyces, Isaria, Fusarium, Mucor* and *Phomopsis* are synonymous to *Geotricum, Cordyceps/Paecylomyces, Fusicola, Rhizomucor* and *Diaporthe*, respectively (Eliskases-Lechner et al., 2011; Gräfenhan et al., 2011; Udayanga et al., 2011; Sun et al., 2020).

The most common genus was *Fusarium* sp., being represented by four isolates that originated from both Puerto Rico and Maryland. Two fungal isolates each of *Cladosporium* sp. *Galactomyces* sp. and *Phomopsis* sp. were represented in our collection. *Cladosporium* sp. was identified in both Puerto Rico and Maryland, while *Galactomyces* sp. and *Phomopsis* sp. isolates were only identified in Maryland or Puerto Rico, respectively. Single isolates were identified for the remaining fungal genera.

### Screening for Entomopathogenic Activity With a Fungus Direct-Exposure Assay Against *An. gambiae* Adults

As an initial screen to identify potential entomopathogenic isolates, our 22 isolated fungi were tested for mosquitocidal activity through a direct exposure assay against adult non-blood-fed females of the major malaria vector *An. gambiae*. This mosquito species is in general more delicate and therefore more susceptible to pathogens than is the more robust arboviral vector *Ae. aegypti* (Beckage et al., 2004; Alkhaibari et al., 2017). In short, adult females were placed directly on the fungus culture agar plate, which was then shaken for 30 s to assure extensive mosquito surface exposure. Sterile BHI agar plates were used as a negative control. The mosquitoes were then transferred to cups, and survival was monitored for 20 days. Fungus-mediated lethality was estimated by calculating the *p*-value with a long-rank Mantel-Cox analysis, and a value of <0.0001 was considered indicative of highly potent entomopathogenic activity. From this first screening, a total of five fungal isolates (isolate 1, two *Galactomyces* sp., *Isaria* sp., *Mucor* 3 sp.) showed potent killing activity against *An. gambiae* females, with these mosquitoes having a significantly (*p* < 0.0001) shorter lifespan than did the non-fungus-exposed mosquitoes (Table 2, Supplementary Figure 1).

**Table 2:** Mortality of *An. gambiae* and *Ae. aegypti* mosquitoes exposed through fungal direct-exposure assay.

***An. gambiae***			
**Fungi**	***P*-value^a^**	**Mortality (days)**	
		**Median^b^**	**Total^c^**
Isolate 1	<0,0001^****^	5	13
Ascomycota	0.6	5	12
*Aureobasidium* sp.	0.6 NS	5	10
*Cercospora* sp.	0.3 NS	3	13
*Cladosporium* 1 sp.	0.02^*^	11	19
*Cladosporium* 2 sp.	0.0006^***^	4	8
*Colletotrichum* sp.	0.0007^***^	10	18
*Fusarium* 1 sp.	0.1 NS	3	11
*Fusarium* 2 sp.	0.8 NS	5	12
*Fusarium* 3 sp.	0.1 NS	9	15
*Fusarium* 4 sp.	0.0009^***^	7	10
*Galactomyces* 1 sp.	<0.0001^****^	5	9
*Galactomyces* 2 sp.	<0.0001^****^	4	9
*Isaria* sp.	<0.0001^****^	3	4
*Mucor* 1 sp.	0,1 NS	8	14
*Mucor* 2 sp.	0.0009^***^	7	12
*Mucor* 3 sp.	<0.0001^****^	1	6
*Penicillium* sp.	0.02^*^	9	17
*Phomopsis* 1 sp.	0.009 NS	11	19
*Phomopsis* 2 sp.	0.2 NS	12	19
*Pleosporales* sp.	0.9 NS	15	20
*Scopulariopsis* sp.	0.1 NS	9	14
Untreated (–ctrl)^d^	/	10	16
*Beauveria bassiana* (+ctrl)^e^	<0.0001^****^	2	3
***Ae. aegypti***			
**Fungi**	***P*** **-value^a^**	**Mortality**	
		**Median^b^**	**Total^c^**
Isolate 1	0,7 NS	14	NA
*Galactomyces* 1 sp.	0,6 NS	14	NA
*Galactomyces* 2 sp.	0,6 NS	14	NA
*Isaria* sp.	<0,0001^****^	4	6
*Mucor* 3 sp.	0,2 NS	17	NA
untreated (–ctrl)^d^	/	14	NA
*Beauveria bassiana* (+ctrl)^e^	<0,0001^****^	1	3

a *P-value calculated by comparing the negative control to each fungus, analyzed by log-rank Mantel-Cox test*;

b *Median indicates the median number of days the mosquitoes survived after exposure*;

c *Total indicates the day when 100% of the mosquitoes had died*;

d *ctrl–, negative control*;

e *ctlr+, positive control; NS, not significant; NA, not achieved, since the mosquitoes survived past day 20. The values in this table were calculated from one replicate*.

* *p < 0.05*,

** *p < 0.01*,

*** *p < 0.001*,

**** *p < 0.0001*.

The median survival of the non-fungus-exposed mosquitoes was 10 days; *Mucor* 3 sp. displayed the most potent lifespan-shortening activity, resulting in a median mosquito survival period of 1 day, followed by *Isaria* sp. (3 days), *Galactomyces* 2 sp. (4 days), and fungal isolate 1 and *Galactomyces* 1 sp. (5 days). Exposure to the positive control *B. bassiana* resulted in a median mosquito survival of 2 days (Table 2). All non-fungus-exposed mosquitoes had died by 16 days, all isolate 1-exposed mosquitoes had died by 13 days, all mosquitoes exposed to either of the two *Galactomyces sp*. isolates had died by 9 days, those exposed to *Isaria* had died by 4 days, and the *Mucor* 3 sp.*-*exposed mosquitoes had died by 6 days. Mosquitoes exposed to the positive control *B. bassiana* were all dead at day 3 (Table 2). Three of the entomopathogenic fungi we discovered, isolate 1, *Isaria* sp., and *Mucor* 3 sp., displayed an intense fungal proliferation/growth on the dead mosquito carcasses. Mosquitoes that had succumbed after *Galactomyces* sp. exposure showed a darker coloration than that of the non-fungus-exposed mosquitoes.

The interesting findings from the screen made us re-evaluate the identification of four of the five potent fungal isolates. Based on the ITS, ribosomal small subunit (SSU) and ribosomal large subunit (LSU)-sequencing data, the phylogenetic trees (Supplementary Files 1–3) and spore photos of the four interesting fungi (Supplementary Figure 2) we were able to suggest a species for four of the five fungal isolates. These are *Galactomyces candidum* 1, *Galactomyces candidum* 2*, Isaria fumosorosea*, and *Mucor hiemalis*.

### Testing for Entomopathogenic Activity Against *Ae. aegypti* Adults

Next, we decided to evaluate the five fungi (isolate 1, *G. candidum* 1, *G. candidum* 2*, I. fumosorosea*, and *M. hiemalis*) that had been shown to be entomopathogenic in the *An. gambiae-*based screen against *Ae. aegypti* using the same direct exposure assay. Of these five fungi, only *I. fumosorosea* showed a highly potent entomopathogenic activity against *Ae. aegypti* that was comparable to the activity of the positive control *B. bassiana*; they both had a *p*-value of <0.0001 (Table 2, Supplementary Figure 1). While the median lifespan of the non-exposed mosquitoes was 14 days, the lifespan for those exposed to *Isaria* sp. or the positive control *B. bassiana* were 4 days and 1 day, respectively. For the *Isaria* sp.-exposed mosquitoes, 100% mortality was reached on day 6, whereas those exposed to the positive control *B. bassiana* died within a 3-day period after exposure (Table 2).

### Determining Entomopathogenic Activity With Spore-Dipping Fungus Exposure Assays Against *An. gambiae* and *Ae. aegypti* Adults

**Figure 2:**
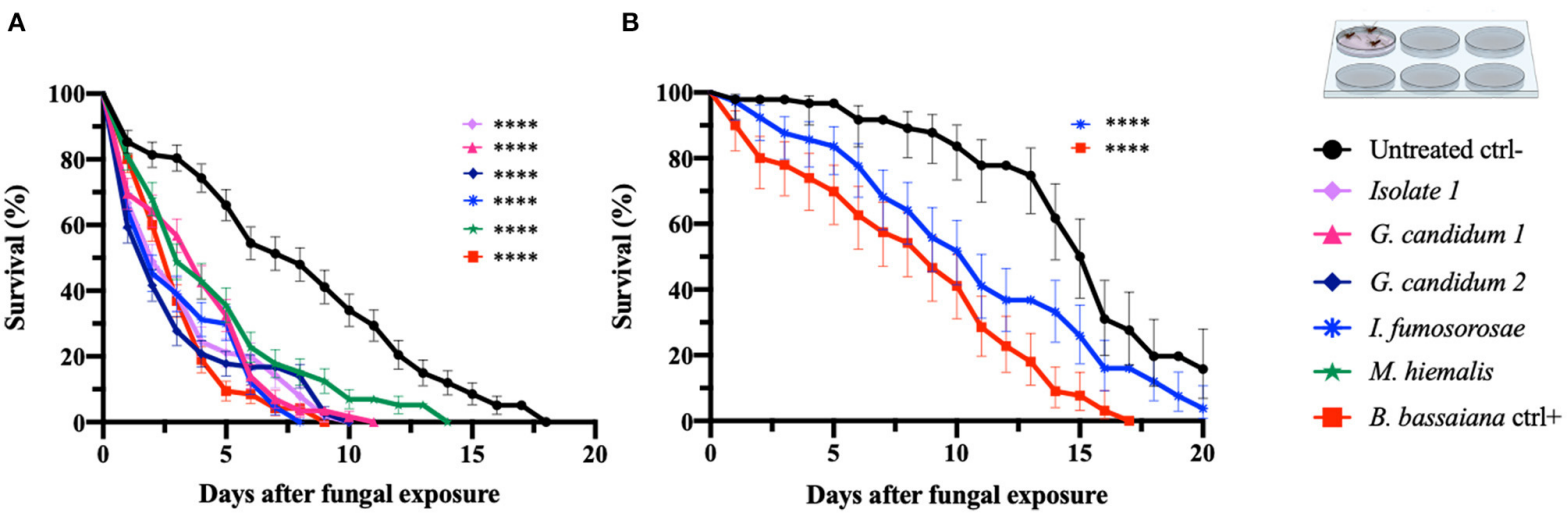
Spore-dipping assays of *An. gambiae* and *Ae. aegypti* mosquitoes. Mosquitoes were exposed via a dipping assay to a spore solution (10^8^ spores/ml) for 1 min, and the mosquito survival was monitored for 20 days. Data are here presented for *An. gambiae* **(A)** and *Ae. aegypti* **(B)**. The fungal species tested on each mosquito species were the ones identified as active in the respective direct-exposure experiment. The graphs represent the average and standard deviation of three biological replicates, *N* = 30 females per replicate. As a negative control, mosquitoes were exposed to a spore-free solution, and as a positive control they were exposed to a spore solution of *B. bassiana*. The statistical significance of the results for each fungus was compared to the negative control and analyzed by log rank-Mantel-Cox test; *p*-values are presented in Table 3. *p < 0.05, **p < 0.01, ***p < 0.001, ****p < 0.0001.

In order to confirm the outcome of the direct-exposure mosquitocidal assays and to further explore the entomopathogenic activities of the five selected fungal isolates, We performed spore-dipping assays against *An. gambiae* and *Ae. aegypti*. This type of assay allowed us to better control the concentration of spores to which mosquitoes were exposed and thereby obtain more reproducible and comparable data. In contrast to the direct exposure assay, in this method the mosquitoes are only exposed to spores and not fungal mycelia, growing hyphae, or possible toxic compounds. We used a spore concentration corresponding to that used in previous studies (Dong et al., 2012; Vivekanandhan et al., 2020b). Adult female mosquitoes were dipped for 1 min into a spore solution, to allow the spores to come in contact with the mosquito surface. As a negative control, mosquitoes were dipped in a non-spore-containing solution. The spore-dipping experiments were performed in triplicate, and the two mosquito species were tested with the respective fungi that had shown a significant (*p* < 0.0001) killing activity in the direct-exposure assay. Five-days-old *An. gambiae* females were tested against isolate 1, *G. candidum* 1 and 2*, I. fumosorosea*, and *M. hiemalis*. All five fungi displayed a strong and significant (*p* < 0.0001) mosquitocidal activity after dipping of the mosquitoes into the spore solution (Table 3, Figure 2A). The median lifespan of the negative control cohort was 8 days, and the mosquitoes exposed to isolate 1, *G. candidum* 2, or *I. fumosorosea* displayed a 2-day median survival time. Mosquitoes exposed to spores of *G. candidum* 1 or *M. hiemalis* showed a median survival time of 4 and 3 days, respectively. Mosquitoes exposed to the positive control *B. bassiana* had a median survival time of 3 days. The spores of these fungal isolates killed all the adults within 6–9 days, depending on the replicates, whereas the non-spore-treated mosquitoes died within 14–18 days in the three biological replicates. These findings were also confirmed with a hazard ratio analysis, which expresses the probability of the death event. For example, isolate 1-exposed mosquitoes that had all succumbed by 6 days after exposure had a hazard ratio of 4.4, indicating that death was 4.4 times more likely after exposure to this fungus than if not exposed. For *G. candidum* 1- and *G. candidum* 2-exposed mosquitoes, the hazard ratios were 3.9 and 4.5, respectively. For *I. fumosorosea*-exposed mosquitoes, which had all died by 6 days after exposure, the hazard value was 5.1, and for *M. hiemalis*-exposed mosquitoes it was 2.5. Mosquitoes exposed to the positive control *B. bassiana* showed a hazard ratio of 5.4 and 100% mortality was observed after 7 days (Table 3). We also screened adult female *Ae. aegypti* against *Isaria* sp. spores, and the results showed a potent reduction of the lifespan (*p* < 0.0001) (Figure 2B), with a median of survival time of 11 days, as compared to 16 days for the negative control and 9 days for the *B. bassiana*-exposed positive control (Table 3).

**Table 3:** Mortality of *An. gambiae* and *Ae. aegypti* mosquitoes exposed to spore solution in the dipping assay.

***An. gambiae***				
**Fungi**	***P*** **-value^a^**	**Mortality (days)**		**Hazard ratio (95% CI)** ^**d**^
		**Median** ^**b**^	**Total + SD** ^**c**^	
Isolate 1	<0.0001^****^	2	6 + 0.8	4.46 (3–6.6)
*Galactomyces candidum* 1	<0.0001^****^	4	8.3 + 2	3.9 (2.7–5.8)
*Galactomyces candidum* 2	<0.0001^****^	2	8.6 + 1.8	4.5 (3.1–6.67)
*Isaria fumosorosae*	<0.0001^****^	2	6.3 + 1.6	5.2 (3,4–7.9)
*Mucor hiemalis*	<0.0001^****^	3	9.6 + 3	2.5 (1.7–3.7)
Untreated (–ctrl)^e^	/	8	14.6 + 2.4	/
*Beauveria bassiana* (+ctrl)^f^	<0.0001^****^	3	7.3 + 1.2	5.4 (3.6–8,1)
***Ae. aegypti***				
**Fungi**	***P*** **-value** ^**a**^	**Mortality (days)**		**Hazard ratio (95% CI)** ^**d**^
		**Median** ^**b**^	**Total + SD** ^**c**^	
Isolate 1	ND	ND	ND	ND
*Galactomyces candidum* 1	ND	ND	ND	ND
*Galactomyces candidum* 2	ND	ND	ND	ND
*Isaria fumosorosea*	<0.0001^****^	11	16.6 + 3.9	2.1 (1.4–3.1)
*Mucor hiemalis*	ND	ND	ND	ND
Untreated (–ctrl)^e^	/	16	NA	/
*Beauveria bassiana* (+ctrl)^f^	<0.0001^****^	9	13.3 + 1.4	4.8 (3.3–7.1)

a *P-value calculated by comparing the negative control to each fungus, analyzed by log-rank Mantel-Cox test*;

b *Median indicates the median number of days the mosquitoes survived after exposure*;

c *Total + SD indicates the day when 100% of the mosquitoes had died, with the corresponding standard error*;

d *Hazard ratio (95% CI), calculated with log-rank Mantel and Haenzel analysis with the corresponding 95% interval of confidence*;

e *ctrl–, negative control*;

f *ctlr+, positive control; NS, not significant; NA, not achieved, since mosquitoes survived past day 20. The values in this table were calculated from three replicates*.

* *p < 0.05*,

** *p < 0.01*,

*** *p < 0.001*,

**** *p < 0.0001*.

All *Ae. aegypti* exposed to *I. fumosorosea* had died by 16 days after exposure, yielding a hazard ratio of 2.1, whereas 57% of the non-spore-exposed control cohort had succumbed by day 20. All mosquitoes had succumbed after exposure to *B. bassiana* spores at 13 days post-treatment, yielding a hazard ratio of 4.8 (Table 3).

### Mosquitocidal Activity of Fungus Culture Filtrates Against *An. gambiae* and *Ae. aegypti* Adults

**Figure 3:**
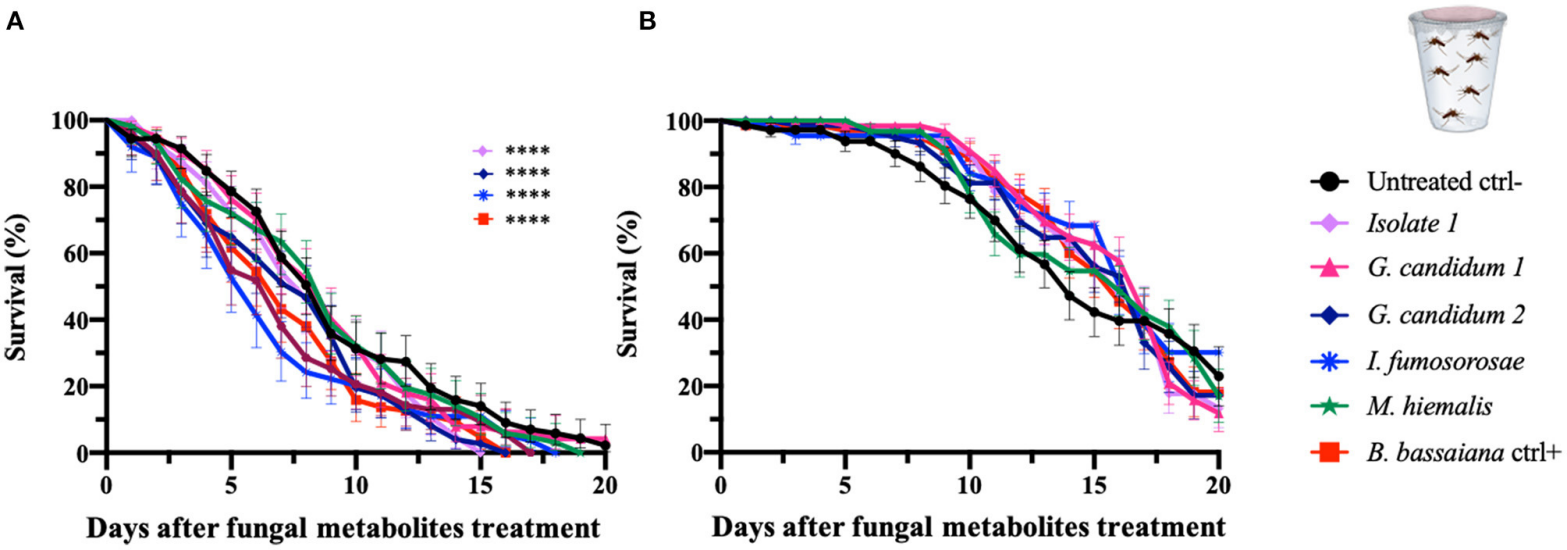
Fungal metabolite assays of *An. gambiae* and *Ae. aegypti* mosquitoes. Mosquitoes were allowed to feed on a sucrose solution containing fungal metabolites (free from spores and mycelium) for 48 h, and the mosquito survival was monitored for 20 days. Data here are presented for *An. gambiae* **(A)** and *Ae. aegypti* **(B)**. The metabolites used in the experiments are derived from the fungi identified as active against *An. gambiae* in the initial screen (direct exposure experiment) and were collected from fungal liquid culture. The graphs represent the average and standard deviation of three biological replicates, *N* = 30 females per replicate. As a negative control, mosquitoes were fed on a metabolite-free solution, and as a positive control on metabolite solutions from *B. bassiana*. The statistical significance of the results for each fungus was compared to the negative control and analyzed by log-rank Mantel-Cox test; *p*-values are presented in Table 4.

To initiate an investigation into the nature of the entomopathogenic activity, we designed assays that would discriminate between killing mechanisms that are mediated by secreted fungus-produced metabolites and those requiring exposure to living fungi. Adult female *An. gambiae* and *Ae. aegypti* were fed on a sucrose solution containing a fungal liquid potato dextrose (PD) culture filtrate that presumably would contain the secreted factors of interest. We examined the five selected mosquitocidal fungi identified in the initial screening against adult *An. gambiae* females. As expected, both mosquito species showed an overall greater survival after ingestion of the fungal culture filtrates than after direct exposure to live fungi or spore solution. In fact, the hazard ratio of *An. gambiae* was between 0.8 and 2.4 and of *Ae. aegypti* mosquitoes <1, for the tested fungal isolates (Table 4, Figure 3).

**Table 4:** Mortality of *An. gambiae* and *Ae. aegypti* mosquitoes exposed to fungal metabolites by feeding assay.

***An. gambiae***				
**Fungi**	***P*** **-value** ^**a**^	**Mortality (days)**		**Hazard ratio (95% CI)** ^**d**^
		**Median** ^**b**^	**Total + SD** ^**c**^	
Isolate 1	0.003^**^	8	14.6 + 0.8	1.3 (1–1.9)
*Galactomyces candidum* 1	0.5 NS	9	16 + 2.8	1 (0.8–1.4)
*Galactomyces candidum* 2	0.006^**^	8	14.3 + 1.6	1.5 (1.3–2.1)
*Isaria fumosorosea*	0.002^**^	6	14.3 + 4.4	1.7 (1.3–2.4)
*Mucor hiemalis*	0.5 NS	9	17.3 + 1.6	1.1 (0.8–1.4)
Untreated (–ctrl)^e^	/	9	18 + 2.8	/
*Beauveria bassiana* (+ctrl)^f^	0.002^**^	7	14.6 + 1.2	1.6 (1.1–2.2)
***Ae. aegypti***				
**Fungi**	***P*** **-value** ^**a**^	**Mortality (days)**		**Hazard ratio (95% CI)** ^**d**^
		**Median** ^**b**^	**Total + SD** ^**c**^	
Isolate 1	0.6 NS	16	NA	0.8 (0.5–1.4)
*Galactomyces candidum* 1	0.5 NS	17	NA	0.8 (0.5–1.4)
*Galactomyces candidum* 2	0.1 NS	17	NA	0.8 (0.5–1.5)
*Isaria fumosorosea*	0.2 NS	17	NA	0.7 (0.4–1.2)
*Mucor hiemalis*	0.6 NS	16	NA	0.89 (0.5–1.4)
Untreated (–ctrl)^e^	/	14	NA	/
*Beauveria bassiana* (+ctrl)^f^	0.5 NS	16	NA	0.85 (0.5–1.4)

a *P-value was calculated by comparing the negative control to each fungus, then analyzed by log-rank Mantel-Cox test*;

b *Median indicates the median number of days the mosquitoes survived after exposure*;

c *Total + SD indicates the day when 100% of the mosquitoes had died, with the corresponding standard error*;

d *Hazard ratio (95% CI) is the hazard ratio with its 95% confidence interval, calculated with log-rank Mantel and Haenzel analysis*;

e *ctrl–, negative control*;

f *ctrl+, positive control; NS, not significant; NA, not achieved, since mosquitoes survived past day 20. The values in this table were calculated from three replicates*.

* *p < 0.05*,

** *p < 0.05*,

*** *p < 0.001*,

**** *p < 0.0001*.

*An. gambiae* mosquitoes that had ingested isolate 1 and *G. candidum* 2 culture filtrates showed a median of survival of 8 days and a significant reduction in the lifespan when compared to untreated mosquitoes (*p* = 0.003 and *p* = 0.006, respectively). *An. gambiae* mosquitoes that fed on the *I. fumosorosea* culture filtrate showed a median survival time of 6 days (*p* = 0.002), and those ingesting the *B. bassiana* culture filtrate showed a median survival of 7 days and a *p*-value of 0.002. Ingestion of *G. candidum* 1 or *M. hiemalis* culture filtrate did not have any effect on *An. gambiae* longevity when compared to the non-culture filtrate-fed control mosquitoes (Table 4, Figure 3A). All *An. gambiae* mosquitoes that were fed on isolate 1, *I. fumosorosea* or *G. candidum* 2 culture filtrates died within 14 days after ingestion, whereas those fed on *G. candidum* 1 or *M. hiemalis* culture filtrates, as well as the untreated mosquitoes, survived up to 16–18 days. The positive control mosquitoes that fed on a *B. bassiana* culture filtrate had all died at 7 days post-feeding (Table 4). As compared to the untreated control mosquitoes, none of the culture filtrates of the five selected fungi affected the survival of *Ae. aegypti* after ingestion, not even the *I. fumosorosea* isolate, which exerted potent killing activity against this mosquito species in the direct-exposure assay (Table 4, Figure 3B). Our results show that some of the fungi do produce and secrete factors that are toxic to the mosquitoes, but the most efficient killing requires exposure to live fungi and presumably involves an active infection process.

## Discussion

The failure of disease control programs resulting from insecticide resistance among mosquito populations highlights the urgent need for new tools for mosquito control, including new insecticides. In the present study, we sampled diverse habitats to find entomopathogenic fungi that could satisfy the requirements for further development into mosquitocidals for use in vector control programs. We collected a total of 36 samples from various habitats and sources in Maryland and Puerto Rico that yielded 76 fungal isolates, 22 of which were successfully cultured under laboratory conditions. We were unsuccessful to maintain all the isolated fungi in the lab for several reasons; some fungi were simply not able to survive in the lab-condition of temperature and humidity, considering that they were isolated in Puerto Rico, some of them could have been in strict symbiosis with other fungi or source like flowers or other plants, some of them were perhaps not able to produce enough spores to propagate.

Analyses of their ribosomal 18S gene, through sequences blasting and through phylogenetic trees generation, revealed that the 22 isolates belonged to 12 different genera: Ascomycota phylum*, Aureobasidium* sp., *Cercospora* sp., *Cladosporium* sp., *Colletotrichum* sp., *Fusarium* sp., *Galactomyces* sp., *Isaria* sp., *Mucor* sp., *Penicillium* sp., *Phomopsis* sp., *Pleosporales* sp. and *Scopulariopsis* sp. Members belonging to the same genus as at least eight of our isolates have previously been shown to possess entomopathogenic properties against various types of insects, including various mosquito species (Ascomycota*, Cladosporium* sp., *Diaporthe* sp., *Fusarium* sp., *Isaria* sp., *Mucor* sp., *Penicillium* sp., and *Scopulariopsis)*. These results further confirm the presence of entomopathogenic fungi in a wide variety of outdoor environments (Lacey and Fransen, 1996; Chandler et al., 1997; Sánchez-Peña et al., 2011). Within the Ascomycota phylum we find the most important entomopathogenic fungi (*Beauveria* spp. and *Metarhizium* spp.) that are used a biopesticides to control mosquitoes (Blanford et al., 2005; Scholte et al., 2005; Hancock, 2009; Hancock et al., 2009; Knols et al., 2010; Farenhorst et al., 2011). Several species of *Cladosporium* sp. (also Ascomycota phylum) have been found to exert pathogenic activity against insects; for example, lab trials showed that a *C. velox* fungus is able to kill *Spodoptera litura* larvae, one of the most important insect pests of agricultural crops in tropical regions of Asia (Singh et al., 2015), and extracts of *C. cladosporioides* have been shown to kill *Aphis gossypii*, which is a major pest of the cotton industry (Elbanhawy et al., 2019). *Diaporthe* sp. filtrates have been shown to have both larvicidal and adulticidal activity against *Ae. aegypti* mosquitoes (Meepagala et al., 2018), and exposure to its conidia has been shown to have pathogenic activity against the strawberry pest *Duponchelia fovealist* (Amatuzzi et al., 2018). The *Fusarium* sp. genus belongs to the Hypocreales order, as do *Beaveria* spp. and *Metarhizium* spp.; species within this genus have been shown to possess entomopathogenic activity against *Tribolium* sp., is an insect pest of stored grains (Chehri, 2017); a *F. verticillioides* isolate that is a grasshopper pathogen (Pelizza et al., 2011); and *F. oxysporum* extracts, which are lethal against larvae and pupae of multiple mosquito vectors such as *An. stephensi, Ae. aegypti*, and *Culex (Cx) quinquefasciatus* (Vivekanandhan et al., 2018a). *Isaria* sp. is a well-known entomopathogenic fungus, already commercialized for the control of different insect species in several countries. The only insecticidal activity of *Mucor hiemalis* has been shown to be correlated with its ability to secrete insecticidal metabolites against the adult stages of the agricultural pests *Bactrocera oleae* and *Ceratitis capitata* (Konstantopoulou et al., 2006). *Penicillium citrum* exerts lethal activity against larvae and adults of *Cx. quinquefasciatus* (Maketon et al., 2014). Finally, *Scopulariopsis* sp. has been found to be lethal to the agricultural pest *Bemisia tabaci* (Niu et al., 2019) (Table 1).

But among these eight already known as EPFs, we have here discovered three novel pathogenic fungi: isolate 1 that was previously unknown and un-characterized, two strains of *Galactomyces candidum* and *Mucor hiemalis*.

Our first screening for mosquitocidal activity was performed through surface exposure of female *An. gambiae* to plated 2-week-old fungal cultures that usually contained between 4 × 10^8^ and 1 × 10^10^ spores/ml, depending on the fungal colony. This assay, that involves the shaking of mosquitoes on the fungal plate ensured an extensive surface exposure to not only spores, but also the fungal mycelia and hyphae and eventual toxic compounds produced by the fungi; however, the somewhat short lifespan of the control mosquitoes suggests that this procedure adversely affected *An. gambiae* viability, maybe due to the mechanical stress during the shaking on the agar plate. Nevertheless, we saw significant differences in survival between the non-exposed mosquitoes and several of the fungus-exposed cohorts, with a stringent statistical evaluation yielding a *p*-value of <0.0001. We identified five fungal isolates that significantly reduced the *An. gambiae* mosquitoes' lifespan and therefore were chosen for further investigation. These five were: isolate 1, *Galactomyces candidum* 1, *Galactomyces candidum* 2*, Isaria fumosorosea*, and *Mucor hiemalis. I. fumosorosea* also displayed a significant (*p* < 0.0001) killing activity against *Ae. aegypti* with the same direct exposure assay.

The isolate 1 fungus that displayed adulticidal activity against *An. gambiae* appears to be a novel mosquitocidal fungus that has not been previously investigated for pathogenic effects on insects, plants, or humans. It did display relatedness to *Aaosphaeria* sp. that has not been addressed with regards to these properties. *G. candidum* is a Saccharomycotina, a yeast-like fungus whose potential pathogenic activities have been investigated in previous studies, and recent laboratory-based experiments have shown killing activity against *Botrytis cinera*, a gray mold causing plant disease (Chen et al., 2018). Studies performed by Chen et al. have suggested that *Galactomyces sp*. is able to produce volatile compounds and chitinase enzymes that inhibit fungal growth. *Galactomyces sp*. is also used in dairy industry (Perkins et al., 2002). *I. fumosorosea* belongs to the Hypocreales order, which includes the well-studied entomopathogenic fungi *Beauveria* spp. and *Metarhizium* spp. *Isaria* sp. has been used as an environmentally friendly pest biocontrol agent in many countries, being registered as a biopesticide in the USA (United States Environmental Protection Agency (EPA)., 2020), in the European Union (European Commission (EU), 2020), and in Brazil (Ministerio de Agricultura, 2020). In China, although this fungus has not been registered as a myco-pesticide, it is widely used to control whiteflies and aphids (Zhang et al., 2016; Xu et al., 2017). Several studies have identified the genus *Isaria* sp. as a source of promising entomopathogenic candidates against adult *Ae. aegypti* and *An. stephensi* as well as *Ae. aegypti* eggs (Luz et al., 2007; Leles et al., 2010*;* Blanford et al., 2012*;* Ramirez et al., 2018). Furthermore, *Isaria* sp. mycelia extracts have been used for the development of silver nanoparticles that are able to kill *Ae. aegypti* and *Cx. quinquefasciatus* larvae (Banu and Balasubramanian, 2014). *M. hiemalis* is a saprophytic species belonging to the Zygomycetes, Mucorales. It is frequently found to infect injured insects (Heitor, 1962), but as far as we know it has not been previously studied with regard to mosquitocidal activity.

We also used a spore-based dipping assay that relied on exposure of mosquitoes to a liquid spore-containing solution to further study the fungi that had shown confirmed entomopathogenic activity in the direct-exposure assay. We reasoned that this method would enable us to expose the mosquitoes to the same concentration of spores in each replicate and that with 1 min of spore exposure we could assess the fungal infection in the mosquitoes, if the fungus was pathogenic. The actual procedure of dipping *An. gambiae* mosquitoes into the spore-free PBS solution seemed to affect mosquito viability, since the control cohort displayed a lifespan of only 14 days. A possible contributing factor to this decreased viability could have been the presence of detergent (Tween-80) that to some degree could permeabilize the external wax layer of the cuticle (Vincent, 2002). However, the generally more robust *Ae. aegypti* mosquitoes were not affected by this treatment. We found that *An. gambiae* exposed to *I. fumosorosea* as well as *M. hiemalis* and *Ae. aegypti* exposed to *I. fumosorosea* through direct exposure, when we assume a more extensive contact with spores occurred, displayed a shorter lifespan than did the mosquitoes exposed to the same fungi in the 10^8^ spores/ml dipping assay. This likely spore dosage-dependent effect was also observed with *B. bassiana* fungus, for which an increased dose of spores was associated with a greater mortality in *Ae. aegypti* mosquitoes (Dong et al., 2012). These results confirm a previous study showing a dosage-dependent entomopathogenic activity of *I. fumosorosea* against *B. tabaci* nymphs (Gao et al., 2017). We also demonstrate here a spore dosage-dependence with regard to *M. hiemalis*. The killing activity caused by the fungal spores did not seem to be exposure method-dependent for all our fungal isolates. For example, exposure to isolate 1 resulted in a 100% mortality of *An. gambiae* by 6 days after dipping in the spore solution, whereas all the mosquitoes of this species treated with the direct-exposure method survived longer and died within 13 days after exposure. This observation highlights the importance of adapting the delivery, or exposure, method to the physiological and morphological characteristics of a particular fungus. However, *An. gambiae* subjected to *G. candidum* 1 in the two different assays displayed an identical median and total mortality time-course, demonstrating a fungal killing mechanism that is not dependent on the mode of exposure. *G. candidum* 2, in contrast, showed higher mortality in the spore-dipping assay than in direct-exposure assay, and this result could potentially be explained by the presence of toxic compounds that, in the spore solution, were able to more efficiently come in contact with the mosquitoes. Mosquitoes, this result was also corroborate with the fungus-culture-filtrate experiment result. The ability of *G. candidum* to produce pathogenic compounds had already been shown in the work of Chen and co-workers, in which they demonstrated the production of volatile toxic compounds and a chitinase by *Galactomyces* that can affect the longevity of adult mosquitoes (Chen et al., 2018).

To investigate whether the mechanism of fungus-mediated killing required interaction with live fungi or was caused by a secreted fungus factor, we exposed *An. gambiae* and *Ae. aegypti* mosquitoes to a fungus-culture filtrate through a sugar meal. Our results indicated that isolate 1, *G. candidum* 2, and *I. fumosorosea* produce mosquitocidal compound(s) against *An, gambiae*. While isolate 1 has not been previously studied with regard to mosquitocidal activity, *G. candidum* and *I. fumosorosea* have previously been shown to produce entomopathogenic factors (Chen et al., 2018). Interestingly, only one of our two *Galactomyces sp*. isolates had this property, thereby demonstrating that different isolates of the same fungal species can produce different metabolites and thus display different entomopathogenic activities. *I. fumosorosea* has previously been shown to produce metabolites with antibacterial, insecticidal, antiviral, and cytotoxic activity (Weng et al., 2019). Ingestion of the fungal-culture filtrates, including that of *B. bassiana*, which is known to produce entomopathogenic metabolites (Bukhari et al., 2011; Mnyone et al., 2011; Vivekanandhan et al., 2018b), had no effect on *Ae. aegypti*. This result likely reflects the greater robustness of this mosquito species and also raises the possibility that some of the fungi-produced toxic factors were in too low concentration to exert an effect. It is also possible that a different route of exposure (i.e., surface exposure) could have yielded a different result with some of the tested fungal culture filtrates.

In summary, in this study we have isolated and characterized five potentially entomopathogenic fungi with activity against two major mosquito disease vectors. Among these fungi, four have never been tested on insect pests before: isolate 1, two strains of *Galactomyces* candidum and *Mucor hiemalis*. We further show and corroborate the particular potential of *Isaria fumosorosea* as a potential biopesticide candidate against various mosquito species.

These fungi merit further investigation as the source of novel biological agents for mosquito control.

## Materials and Methods

### Ethics Statement

This study was carried out in strict accordance with the recommendations in the Guide for the Care and Use of Laboratory Animals of the National Institutes of Health. The protocol was approved by the Animal Care and Use Committee of the Johns Hopkins University.

### Sample Collection

Organic material with potential fungus growth was collected from different outdoor environments in Maryland and in Puerto Rico. Each sample was collected with sterile forceps, placed in a 1.5-ml tube with 200 μl of sterile 1X PBS and kept at 4°C until further use.

### Culture and Isolation of Fungi

Each sample was manually homogenized with a pestle and plated on brain heart infusion (BHI) agar containing 50 μg/ml of chloramphenicol and left at room temperature until fungal growth was observed. From each plate, every different fungus that grew was single-subcultured until the fungal culture was axenic. All the fungi were maintained on BHI except for *Aaosphaeria arxii*, which was maintained on Sabouraud dextrose agar medium. *Beauveria bassiana* strain 80.2, kindly provided by Dr. Silverman, was used as a positive control and was maintained under the same conditions as the other fungi.

### Identification of Isolated Fungi

DNA from 0.5 cm of fungal culture was extracted with a DNeasy Blood and Tissue Kit (Qiagen) according to the manufacturer's instructions. The fungal biomass was homogenized for 2 min in 200 μl of lysis buffer using 0.5-mm sterile glass beads. Two pairs of degenerate fungal primers were used to amplify the ITS ribosomal 18S region: ITS1 forward and ITS3 forward (ITS1 5′-CTHGGTCATTTAGAGGAASTAA, ITS3 5′-FAHCGATGAAGAACRYAG), and ITS4 reverse (ITS4 5′-RTCCTCCGCTTWTTGWTWTGC) (Toju et al., 2012). One primer pair was used to amplify the 18S small subunit-SSU (NS1-F 5′-GTAGTC ATATGCTTGTCTC, NS4-R 5′- CTT CCGTCAATTCCTTTAAG) and one pair for the 28S large sub unit LSU (LROR-F 5′-ACCCGCTGAACTTAAGC, LR6-R 5′-CGCCAGTTCTGCTTACC) (Raja et al., 2017).

The target amplicon was column-purified using a ZYMO kit and sent to Quintara Biosciences for Sanger sequencing, and the returned sequence was blasted in the NCBI database against fungus taxa for species identification. The ITS-18S sequences were also used to generate phylogenetic trees using the Bootstrap and Neighbor-joining statistical method with the p-distance model through the MEGAX software.

A morphological analysis of spores from a selection of fungal isolates was performed. Spores were obtained from 2 week old fungal cultures. PBS+ 0,02% tween 80 was added to the agar plate and the fungi was scraped loose, filtered through glass wool and transferred to Eppendorf tubes. Samples were centrifuged at 5,000 rpm for 5 min to remove any remaining debris. The supernatant was analyzed under bright field microscope and photos of spores were acquired at 40x magnification using a Leica DM2500 microscope and a DFC310 FX Digital Color Camera (Leica Microsystems).

### Mosquito Rearing

Rockefeller strain *Ae. aegypti* and Keele strain *An. gambiae* were maintained in an insectary chamber at 27°C and 80% humidity on a 12-h light/dark cycle according to standard rearing procedures. The larvae were fed on sterile pulverized fish food, and adult mosquitoes were provided with a 10% sucrose solution. The mosquitoes for the experiments were kept in cups inside a large incubator provided with the same environmental conditions as in the insectary chamber described above.

### Fungal Direct Exposure Assays

To assess the first screening experiments, 30 not blood-fed females, each 3–5 days old, were tested against fungi. Mosquitoes were cold-anesthetized, placed directly into an agar plate containing a 2-week-old fungal culture, and shaken for 30 s. Here, the mosquitoes are exposed to a complex content of fungal spores, fungal mycelia, the growing hyphae, and possible toxic compounds. Thereafter, the mosquitoes were placed in cups with 10% sucrose solution; to avoid bacterial and fungal growth, the sucrose paper was changed three times per week. A plate with *B. bassiana* was used as a positive control, and sterile BHI agar medium was used as a negative control.

All the direct exposure experiments were done once, and mosquito survival was checked daily for 20 days. For logistic reasons, the 22 fungal isolates against *An. gambiae* were tested at three different times, always including the positive and negative controls, and *p*-values were calculated against the respective negative controls.

### Fungal Dipping-Based Exposure Assays

To confirm the mosquitocidal potential of the five pathogenic fungi, a fungal spore exposure assay was performed on 30 female *An. gambiae* and 30 female *Ae. aegypti* mosquitoes, only against those fungi for which a *p*-value of <0.0001 was obtained from direct exposure. The fungal spore exposure was done using purified spores and the dipping procedure (Dong et al., 2012), with the mosquitoes exposed to a solution of 10^8^ spores/ml. The spores were collected from 2-week-old fungal plate cultures, counted with a Neubauer hemocytometer, and adjusted to 10^8^ spores/ml. In brief, mosquitoes were placed, back first, into 2 ml of the spore solution for 1 min. Only the back of the mosquito came in contact with the spore solution. *B. bassiana* spore solution was used as a positive control, and 1X PBS with 0.02% tween-80, used to collect spores, was used as a negative control. Mosquito sugar papers were changed three times per week. The dipping experiments were done in triplicate, and mosquito survival was checked daily for 20 days.

### Fungus Culture Filtrate-Feeding Assays

To discriminate the fungal killing activity as a result of fungal proliferation on mosquito bodies from the potential presence of toxic compounds, 30 females of *An. gambiae* and *Ae. aegypti* mosquitoes were exposed to fungal metabolites through a feeding assay. Fungal metabolites were collected from a 2-week-old fungal liquid culture (potato dextrose medium) (Vandermolen et al., 2013) by filtration through a 0.2-μm filter, then added to a 10% sucrose solution to give a final concentration of 5% sucrose. Metabolites produced by *B. bassiana* were used as a positive control, and filtered potato dextrose broth with sucrose was used as a negative control. Mosquitoes were fed on the metabolite/sucrose solution for 48 h, and then the solution was replaced with 10% sucrose. The sucrose papers were changed three times per week as for all other experiments. To confirm that the mosquitoes had died as a consequence of the activity of the metabolites and not because of fungal growth, dead mosquitoes were checked each week under an optical microscope to confirm the absence of fungal proliferation on the mosquito bodies. These assays were run in triplicate, and the mosquito survival rate was checked daily for 20 days.

### Statistical Analysis

Mortality was expressed as a median, and the final day of mosquito survival and the hazard ratio were calculated by Manten and Haenzel analysis. The statistical significance of survival curves was set to the conventional α <0.05 level, calculated with a long-rank Mantel-Cox analysis and using Graphpad Prism software, version 8.

## Data Availability Statement

The original contributions presented in the study are included in the article/Supplementary Material, further inquiries can be directed to the corresponding author.

## Ethics Statement

The animal study was reviewed and approved by Animal Care and Use Committee of the Johns Hopkins University.

## Author Contributions

AA and CE performed the experiments. All authors conceived experiments, analyzed the data obtained, and wrote the manuscript.

## Conflict of Interest

The authors declare that the research was conducted in the absence of any commercial or financial relationships that could be construed as a potential conflict of interest.

## Publisher's Note

All claims expressed in this article are solely those of the authors and do not necessarily represent those of their affiliated organizations, or those of the publisher, the editors and the reviewers. Any product that may be evaluated in this article, or claim that may be made by its manufacturer, is not guaranteed or endorsed by the publisher.
