# Supplementary material for: Discovery of Novel Entomopathogenic Fungi for Mosquito-Borne Disease Control
Source: Front Fungal Biol. 2021 Jul 27;2:637234. doi: 10.3389/ffunb.2021.637234 (PMC10512396; doi:10.3389/ffunb.2021.637234)

Supplementary Table 1: List of sequence primers and combination primers, used for ITS, LSU and SSU amplification.

Gene	Primer Name	Sequence
ITS	ITS1 Forward	CTHGGTCATTTAGAGGAASTAA
ITS	ITS3 Forward	AHCGATGAAGAACRYAG
ITS	ITS4 Reverse	TCCTCCGCTTWTTGWTWTGC
LSU	LROR Forward	ACC CGC TGA ACT TAA GC
LSU	LR6 Reverse	CGC CAG TTC TGC TTA CC)
SSU	NS1 Forward	GTAGTC ATATGCTTGTCTC
SSU	NS4 Reverse	ACC CGC TGA ACT TAA GC
	**Fungal isolate**	**Primers used for ITS amplification and sequencing**
	Isolate 1	ITS3-ITS4
	Ascomycota	ITS1-ITS4
	Aureobasidium	ITS3-ITS4
	Cercospora	ITS1-ITS4
	Cladosporium	ITS3-ITS4
	Cladosporium 2	ITS1-ITS4
	Colletotrichum	ITS3-ITS4
	Fusarium 1	ITS3-ITS4
	Fusarium 2	ITS1-ITS4
	Fusarium 3	ITS1-ITS4
	Fusarium 4	ITS1-ITS4
	Galactomyces 1	ITS1-ITS4
	Galactomyces 2	ITS1-ITS4
	Isaria	ITS3-ITS4
	Mucor 1	ITS3-ITS4
	Mucor 2	ITS1-ITS4
	Mucor 3	ITS1-ITS4
	Penicillium	ITS1-ITS4
	Phomopsis 1	ITS3-ITS4
	Phomopsis 2	ITS1-ITS4
	Pleosporales	ITS3-ITS4
	Scopulariopsis	ITS3-ITS4

Supplementary Figure 1. Mosquitoes were directly exposed to a 2-weeks-old fungal plate and shaken for 30 seconds, the mosquito survival was monitored for 20 days. Data is here presented for the species *An. gambiae* (A) and *Ae. aegypti* (B). As a negative control, mosquitoes were shaken on a sterile BHI agar and as a positive control on a *B. bassiana* plate. These graphs represent one replicate. Statistical significance of survival was calculated for each fungus compared to its negative control using the long rank Mantel Cox test and p values are presented in Table1.

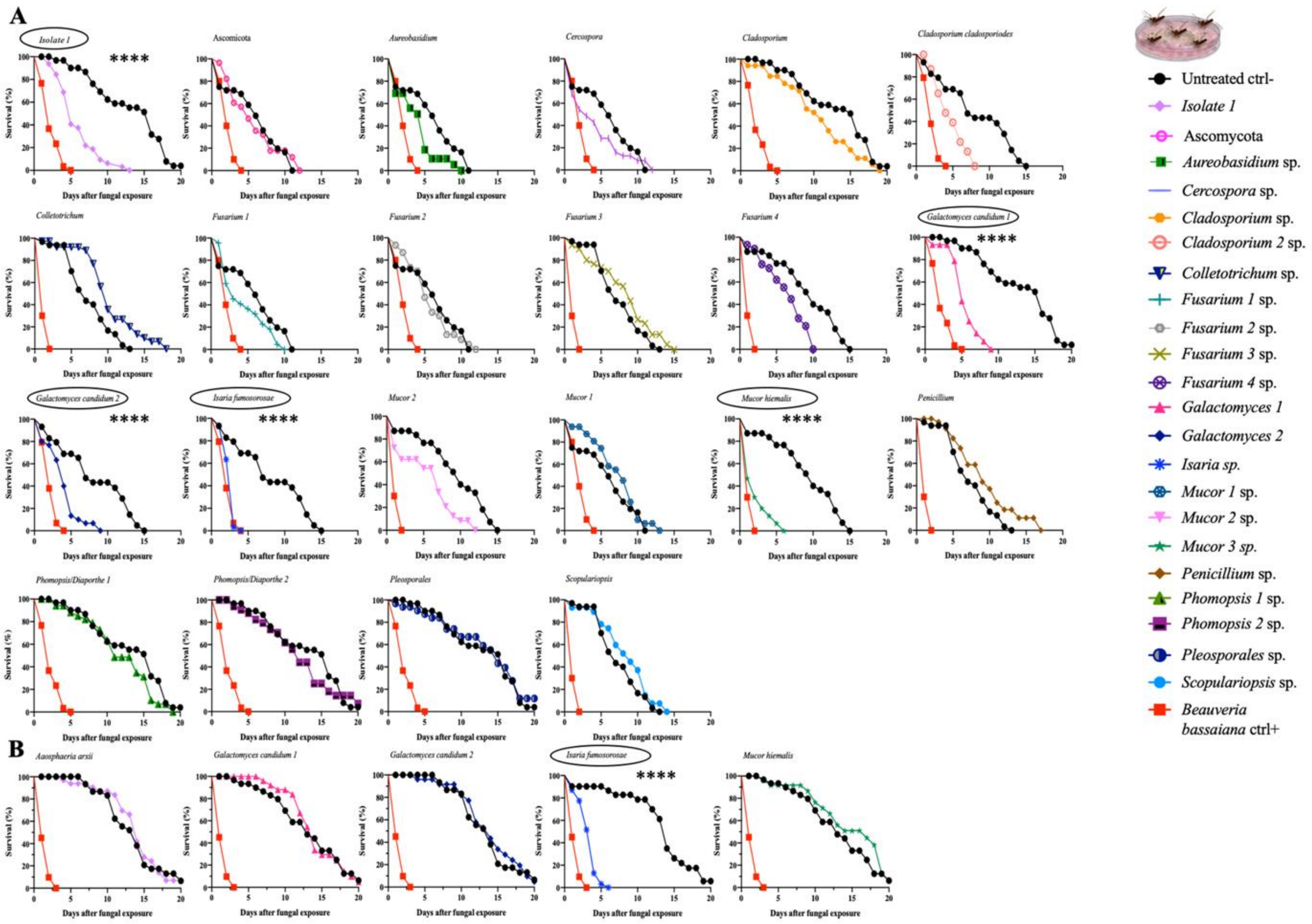

Supplementary Figure 2. Spore pictures of the 5 most potent fungi.

Spores of the 5 most potent fungi (*Galactomyces candidum* 1 and 2, *Isaria fumusorosea, Mucor hiemalis* and isolate 1) were obtained from two week old fungal cultures. PBS was added to the agar plate and the fungi was scraped loose, filtered through glass wool and transferred to Eppendorf tubes. Samples were centrifuged and the supernatants were analysed under bright field microscope. Photos of spores were taken at a 40x magnification using a Leica DM2500 microscope and a DFC310 FX Digital Color Camera (Leica Microsystems).

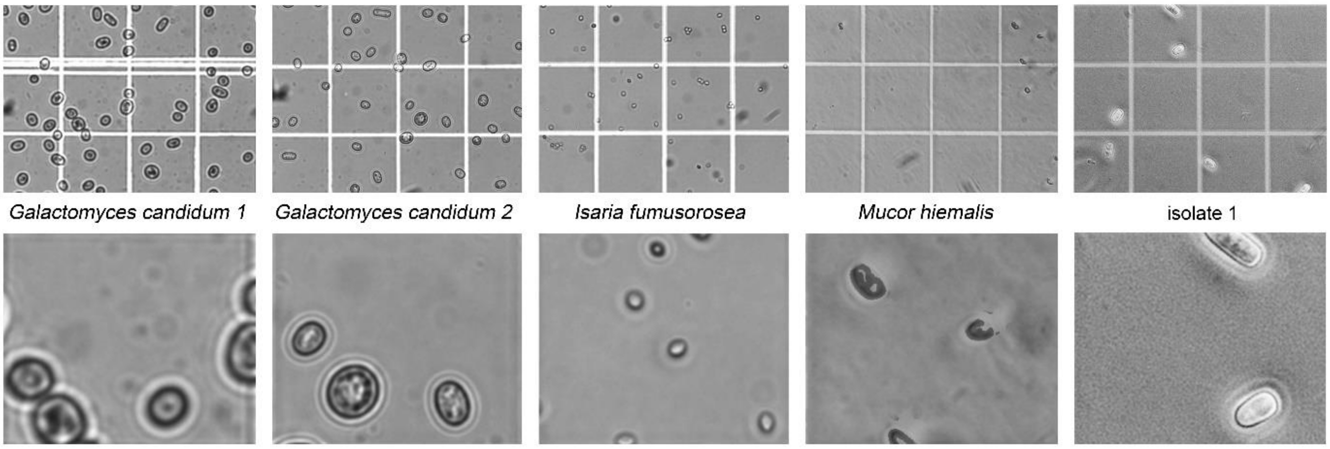

Supplementary file 1: ITS sequences of the 22 fungal isolates used for Blast search and the generation of phylogenetic trees

Isolate 1

GATCATCGAATCTTTGAACGCACATTGCGCCCCTCGGTATTCCGTGGGGCATGCC
TGTTCGAGCGTCATTTAACCCCTCAAGCCTAGCTTGGTATTGGGTGCTTGTCCCGC
CTCTCGCGCGGCGACTCACCTCAAAGTCATTGGCAGCCCGCATCTCGCCGGCCGT
GAGCGCAGCACAGACGCGCTCTTGGCAACGACGGATCGGCTCTCCAAAAGCTTA
TTTCAACCACTGACCTCGGATCAGGTAGGGATACCCGCTGAACTTAAGCATAACA
AAAAAGCGGAGGAA

Isolate 2: Ascomycota

TGCGGAGGGATCATTACTGAGTCTATCTCAAAACCCTTTGTGAACCTTTATAACC
TGTTGCTTCGGCGGCGCGGCCCCCGGGTCGTGCCCGCCGGTATCATCAGAATCTC
TGTTCGAACCCGACGATACTTCTGAGTGTTCTAAGCGAACTGTTAAAACTTTCAA
CAACGGATCTCTTGGCTCCAGCATCGATGAAGAACGCAGCGAAACGCGATATGT
AATGTGAATTGCAGAATTCAGTGAATCATCGAATCTTTGAACGCACATGGCGCCT
TCCAGTATCCTGGGAGGCATGCCTGTCCGAGCGTCGTTTCAACCCTCGAGCCCTC
GTGGCCCGGCGTTGGGGATCTGCCCAGGCAGGCCCCGAAAACCAGTGGCGGACC
CGTCCGGGACCTCTCCTTGCGTAGTAGCATCAGCCTCGCATCGGGAGCCGGCGGG
CCTTCCGGCCTCTAAACCCCCCACAAGTCCGCTCCGGCGGCATCAAGGTTGACCT
CGGATCAGGTAGGAATACCCGCTGAACTTAAGCATATCAAAAAGCGGAGGAA

Isolate 3: Aureobasidium sp.

ATCATCGAATCTTTGAACGCACATTGCGCCCCTTGGTATTCCGAGGGGCATGCCT
GTTCGAGCGTCATTACACCACTCAAGCTATGCTTGGTATTGGGTGCCGTCCTTAG
TTGGGCGCGCCTTAAAGACCTCGGCGAGGCCTCACCGGCTTTAGGCGTAGTAGA
ATTTATTCGAACGTCTGTCAAAGGAGAGGACTTCTGCCGACTGAAACCTTTTATT
TTTTCTAGGTTGACCTCGGATCAGGTAGGGATACCCGCTGAACTTAAGCATATCA
AAAAGCGGAGGAA

Isolate 4: Cercospora sp.

GTGAGGGCCTTCGGGCTCGACCTCCAACCCTTTGTGAACACAACTTGTTGCTTCG
GGGGCGACCCTGCCGTTTCGACGGCGAGCGCCCCCGGAGGCCTTCAAACACTGC
ATCTTTGCGTCGGAGTTTAAGTAAATTAAACAAAACTTTCAACAACGGATCTCTT
GGTTCTGGCATCGATGAAGAACGCAGCGAAATGCGATAAGTAATGTGAATTGCA
GAATTCAGTGAATCATCGAATCTTTGAACGCACATTGCGCCCCTTGGTATTCCGA
GGGGCATGCCTGTTCGAGCGTCATTTCACCACTCAAGCCTCGCTTGGTATTGGGC
GCCGCGGTGTTCCGCGCGCCTCAAAGTCTCCGGCTGAGCTGTCCGTCTCTAAGCG
TTGTGATTTCATTAATCGCTTCGGAGCGCGGGCGGTCGCGGCCGTTAAATCTTTT
ACAAGGTTGACCTCGGATCAGGTAGGGATACCCGCTGAACTTAAGCATAACAAT
AAGCGGAGGA

Isolate 5: Cladosporium 1 sp.

CATGCCTGTTCGAGCGTCATTTCACCACTCAAGCCTCGCTTGGTATTGGGCAACG
CGGTCCGCCGCGTGCCTCAAATCGACCGGCTGGGTCTTCTGTCCCCTAAGCGTTG
TGGAAACTATTCGCTAAAGGGTGCTCGGGAGGCTACGCCGTAAAACAAACCCAT
TTCTAAGGTTGACCTCGGATCAGGTAGGGATACCCGCTGAACTTAAGCATATCAA
TAAGCGGAGGAA

Isolate 6: Cladosporium 2 sp.

TACAAGTGACCCCGGTCTAACCACCGGGATGTTCATAACCCTTTGTTGTCCGACT
CTGTTGCCTCCGGGGCGACCCTGCCTTCGGGCGGGGGCTCCGGGTGGACACTTCA
AACTCTTGCGTAACTTTGCAGTCTGAGTAAACTTAATTAATAAATTAAAACTTTT
AACAACGGATCTCTTGGTTCTGGCATCGATGAAGAACGCAGCGAAATGCGATAA
GTAATGTGAATTGCAGAATTCAGTGAATCATCGAATCTTTGAACGCACATTGCGC
CCCCTGGTATTCCGGGGGGCATGCCTGTTCGAGCGTCATTTCACCACTCAAGCCT
CGCTTGGTATTGGGCAACGCGGTCCGCCGCGTGCCTCAAATCGACCGGCTGGGTC
TTCTGTCCCCTAAGCGTTGTGGAAACTATTCGCTAAAGGGTGTTCGGGAGGCTAC
GCCGTAAAACAACCCCATTTCTAAGGTTGACCTCGGATCAGGTAGGGATACCCG
CTGAACTTAAGCATATCAAAAAGCGGAGGAA

Isolate 7: Colletotricum sp.

GAATCTTTGAACGCACATTGCGCCCGCCAGCATTCTGGCGGGCATGCCTGTTCGA
GCGTCATTTCAACCCTCAAGCACCGCTTGGCGTTGGGGCCCTACGGCTTCCGTAG
GCCCCGAAATACAGTGGCGGACCCTCCCGGAGCCTCCTTTGCGTAGTAACATACC
ACCTCGCACTGGGATCCGGAGGGACTCCTGCCGTAAAACCCCCCAATTTATCAAG
GTTGACCTCGGATCAGGTAGGAATACCCGCTGAACTTAAGCATATCAAAAAGCG
GAGGA

Isolate 8: Fusarium 1 sp.

CTACTACGCTATGGAAGCTCGACGTGACCGCCAATCAATTTGGGGAACGCGATTT
GACTCGCGAGTCCCAACACCAAGCTGGGCTTGAGGGTTGAAATGACGCTCGAAC
AGGCATGCCCGCCAGAATACTGGCGGGCGCAATGTGCGTTCAAAGATTCGATGA
TTCACTGAATTCTGCAATTCACATTACTTATCGCATTTTGCTGCGTTCTTCATCGA
TA

Isolate 9: Fusarium 2 sp.

TTTACAACTCCCAAACCCCTGTGAACATACCTATCGTTGCTTCGGCGGGTCGCCC
CAGCGCCTTCGGGCCTGGACCCAGGCGCCCGCCGGAGGACCCAAACTCTTGTTTT
TTTGAGTATCTTCTGAGTAAACAAGCAAATAAATTAAAACTTTCAACAACGGATC
TCTTGGTTCTGGCATCGATGAAGAACGCAGCGAAATGCGATAAGTAATGTGAATT
GCAGAATTCCGTGAATCATCGAATCTTTGAACGCACATTGCGCCCGCCAGTATTC
TGGCGGGCATGCCTGTTCGAGCGTCATTTCAACCCTCAAGCCCCCGGGCTTGGTG
TTGGGGCTCGGCCCGTCCCTAGCGGACGCGCCGTCCCCGAAACCTAGTGGCGGTC
TCGCTGTAGCCTCCTCTGCGTAGTAGCTAACACCTCGCAACGGGAACGCAGCGCG
GCCACGCCGTTAAACCCCCCACTTCTGAAGGTTGACCTCGGATCAGGTAGGAATA
CCCGCTGAACTTAAGCATATCAAAAAGCGGAGGA

Isolate 10: Fusarium 3 sp.

CCAATTGTTGCCTCGGCGGATCAGCCCGCTCCCGGTAAAACGGGACGGCCCGCC
AGAGGACCCCTAAACTCTGTTTCTATATGTAACTTCTGAGTAAAACCATAAATAA
ATCAAAACTTTCAACAACGGATCTCTTGGTTCTGGCATCGATGAAGAACGCAGCA
AAATGCGATAAGTAATGTGAATTGCAGAATTCAGTGAATCATCGAATCTTTGAAC
GCACATTGCGCCCGCCAGTATTCTGGCGGGCATGCCTGTTCGAGCGTCATTTCAA
CCCTCAAGCCCCCGGGTTTGGTGTTGGGGATCGGCGAGCCCTTGCGGCAAGCCG
GCCCCGAAATCTAGTGGCGGTCTCGCTGCAGCTTCCATTGCGTAGTAGTAAAACC
CTCGCAACTGGTACGCGGCGCGGCCAAGCCGTTAAACCCCCAACTTCTGAATGTT
GACCTCGGATCAGGTAGGAATACCCGCTGAACTTAAGCATATCAAAAAGCGGAG
GAA

Isolate 11: Fusarium 4 sp.

TTACCGAGTTTACAACTCCCAAACCCCTGTGAACATACCTTAATGTTGCCTCGGC
GGATCAGCCCGCGCCCCGTAAAACGGGACGGCCCGCCAGAGGACCCAAACTCTA
ATGTTTCTTATTGTAACTTCTGAGTAAAACAAACAAATAAATCAAAACTTTCAAC
AACGGATCTCTTGGTTCTGGCATCGATGAAGAACGCAGCAAAATGCGATAAGTA
ATGTGAATTGCAGAATTCAGTGAATCATCGAATCTTTGAACGCACATTGCGCCCG
CTGGTATTCCGGCGGGCATGCCTGTTCGAGCGTCATTTCAACCCTCAAGCCCTCG
GGTTTGGTGTTGGGGATCGGCTCTGCCTTCTGGCGGTGCCGCCCCCGAAATACAT
TGGCGGTCTCGCTGCAGCCTCCATTGCGTAGTAGCTAACACCTCGCAACTGGAAC
GCGGCGCGGCCATGCCGTAAAACCCCAACTTCTGAATGTTGACCTCGGATCAGGT
AGGAATACCCGCTGAACTTAAGCATATCAAAAAGCGGAGGAGGGGCCTCCCAAA
TAAATCATTCTTAAATTTGATCTGAAATCAGGCGGGATTACCCGCTGAACTTAAG
CATATCAATAAGCGGAGGA

Isolate 12: Galactomyces 1 sp.

ATCATTATGAATTATTAATATTTGTGAATTTACCACAGCAAACAAAAATCATACA
ATCAAAACAAAAATAATTAAAACTTTTAACAATGGATCTCTTGGTTCTCGTATCG
ATGAAGAACGCAGCGAAACGCGATATTTCTTGTGAATTGCAGAAGTGAATCATC
AGTTTTTGAACGCACATTGCACTTTGGGGTATCCCCCAAAGTATACTTGTTTGAG
CGTTGTTTCTCTCTTGGAATTGCTTTGCTCTTCTAAAATTTCGAATCAAATTCGTTT
GAAAAACAACACTATTCAACCTCAGATCAAGTAGGATTACCCGCTGAACTTAAG
CATATCAATAAGCGGAGGAA

Isolate 13: Galactomyces 3 sp.

ATCATTATGAATTAATAATATTTGTGAATTTACCACAACAAACATCAATCATACA
ATCAATAATTAAAAAATTAAAACTTTTAACAATGGATCTCTTGGTTCTCGTATCG
ATGAAGAACGCAGCGAAACGCGATATTTCTTGTGAATTGCAGAAGTGAATCATC
AGTTTTTGAACGCACATTGCACTTTGGGGTATCCCCCAAAGTATACTTGTTTGAG
CGTTGTTTCTCTCTTGGAATTGCTTTGCTCTTCTAAAATTTCGAATCAAATTCGTTT
GAAAAACAACACTATTCAACCTCAGATCAAGTAGGATTACCCGCTGAACTTAAG
CATATCAATAAGCGGAGGAA

Isolate 14: Isaria sp.

ATCATCGAATCTTTGAACGCACATTGCGCCCGCCAGCATTCTGGCGGGCATGCCT
GTTCGAGCGTCATTTCAACCCTCGACGTCCCCCGGGACGTCGGCCTTGGGGACCG
GCAGCACCCCGCCGGCCCTGAAATGGAGTGGCGGCCCGTCCGCGGCGACCTCTG
CGAAGTACTACAGCTCGCACCGGAAACCCGACGCGGCCCCGCCGTGAAACCCCC
AACTCTGAACGTTGACCTCGGATCAGGTAGGACTACCCGCTGAACTTAAGCATAT
CAAAAAGCGGAGGAA

Isolate 15: Mucor 1 sp.

CCTGTTTCAGTATCAACAACAACCCACATCCACAATTTTGTTGTGAATGGAAGTG
AGAGTATCGATGTAAAAATTGAACTCTTTAAAACTATTAGGCCTGAACTATTGTT
CTATTAGCCTGAACATTTTTTTAATATAAAGGAATGCTCTAGTTAAAAGACTATC
TTGGGGGCCTCCCAAATAAATCATTTTTTAAACTTGATCTGAAATCAGGTGGGAT
TACCCGCTGAACTTAAGCATATCAATAAGCGGAGGAA

Isolate 16: Mucor 2 sp.

TTTTATTTATGGGAGGCCCCTAAATAATAAGCTTTTTTTTAAAACATTTGAACCTA
GAGCTTTTCCTTTATATTAAAAAAAAAGTTCAGGCAAATTTAAAAAACTAAAATT
TCAGGCCTAATATTTTTAAAGAGAACGGCGAACAAATAAATGTCAACCGTAAAA
GTACTCTCAATTCCATCAGCAAAAATCTATCTAAAAAAAAAATAAATTTTTGGAT
GTGGGGGGTTTCTGATACTGAAACAGGCGTGCTCATTGGAATACCAATGAGCGC
AAGTTGCGTTCAAAGACTCGATGATTCACTGAATATGCAATTCACACTAGTTATC
GCACTTTGCTACGTTCTTCATCGATGCGAGAACCAAGAGATCCGTTGTTAAAAGT
TGTTTTTATAGATTTCTTAGGTCTATGTTACAATATTTTATTCTGAATTCTTTTGG

Isolate 17: Mucor 3 sp.

CGGAGGATCATTAAATAATTTAGATGGCCTTTGCTAGTTTTCTAGCGAATGGTTC
ATTCTTTTTTACTGTGAACTGTTTTAATTTTTCAGCGTCTGAGGAATGTCTTTTAGC
CATAGGGATAGGCTACTAGAATGTTAACCGAGCTGAAAGTCAGGCTTAGGCCTG
GTATCCTATTAATTATTTACCAAAAGAATTCAGTATTATAATTGTAACATAAGCG
TAAAAAACTTATAAAACAACTTTTAACAACGGATCTCTTGGTTCTCGCATCGATG
AAGAACGTAGCAAAGTGCGATAACTAGTGTGAATTGCATATTCAGTGAATCATC
GAGTCTTTGAACGCAACTTGCGCTCAATGGTATTCCATTGAGCACGCCTGTTTCA
GTATCAAAAACACCCCACATTCATAATTTTGTTGTGAATGGAAATGAGAGTTTCG
GCTTTATTGCTGAATTCTTTAAAATTATTAGGCCTGAACTATTGTTCTTTCTGCCT
GAACATTTTTTTAATATAAAGGAATGCTCTAGTAAAAAGACTATCTCTGGGGCCT
CCCAAATAAATCATTCTTAAATTTGATCTGAAATCAGGCGGGATTACCCGCTGAA
CTTAAGCATATCAA

Isolate 18: Penicillium sp.

TTTAACGAACCTTTGTTGCTTCGGCGGGCCCGCCTCACGGCCGCCGGGGGGCTTC
TGCCCCCGGGCCCGCGCCCGCCGAAGACCCCTGTGAACGCTGTCTGAAGTATGC
AGTCTGAGAAACTAGCTAAATTAGTTAAAACTTTCAACAACGGATCTCTTGGTTC
CGGCATCGATGAAGAACGCAGCGAAATGCGATAACTAATGTGAATTGCAGAATT
CAGTGAATCATCGAGTCTTTGAACGCACATTGCGCCCTCTGGTATTCCGGAGGGC
ATGCCTGTCCGAGCGTCATTGCTGCCCTCAAGCACGGCTTGTGTGTTGGGCCCCC
GTCCCCCCCTCGTCGGGGGGACGGGCCCGAAAGGCAGCGGCGGCACCGCGTCCG
GTCCTCGAGCGTATGGGGCTTCGTCACCCGCTCTTGTAGGCCCGGCCGGCGCCAG
CCGACCCCAACCCTAAATTTTTTTCAGGTTGACCTCGGATCAGGTAGGGATACCC
GCTGAACTTAAGCATATCAATAAGCGGAGGA

Isolate 19: Phomopsis 1 sp.

ATCTTTGAACGCACATTGCGCCCTCTGGTATTCCGGAGGGCATGCCTGTTCGAGC
GTCATTTCAACCCTCAAGCATTGCTTGGTGTTGGGGCACTGCTTCTAACGAAGCA
GGCCCTGAAATCTAGTGGCGAGCTCGCCAGGACCCCGAGCGCAGTAGTTAAACC
CTCGCTCTGGAAGGCCCTGGCGGTGCCCTGCCGTTAAACCCCCAACTTCTGAAAA
TTTGACCTCGGATCAGGTAGGAATACCCGCTGAACTTAAGCATATCAAAAAGCG
GAGGAA

Isolate 20: Phomopsis 2 sp.

GCCTGTTCGAGCGTCATTTCAACCCTCAAGCATTGCTTGGTGTTGGGGCACTGCT
TCTAACGAAGCAGGCCCTGAAATCTAGTGGCGAGCTCGCCAGGACCCCGAGCGC
AGTAGTTAAACCCTCGCTCTGGAAGGCCCTGGCGGTGCCCTGCCGTTAAACCCCC
AACTTCTGAAAATTTGACCTCGGATCAGGTAGGAATACCCGCTGAACTTAAGCAT
ATCAATAAGCGGAGGA

Isolate 21: Pleosporales sp.

CATGCCTGTTCGAGCGTCATTTGTACCTTCAAGCTTTGCTTGGTGTTGGGCGTTTT
GTCTTTTTATTTTCTAAATAGACTCGCCTCAAAGTAATTGGCAGCCAGTGTTTTTG
GTAGTAAGCGCAGCACATTTTGCGTCTTCGTCTTCAAACAGCGGCATCCACAAAG
CCTCTTTCTCACTTTTGACCTCGGATCAGGTAGGGATACCCGCTGAACTTAAGCA
TATCAAAAAGCGGAGGAA

Isolate 22: Scopulariopsis sp.

GGATCTACTACGCAGGGGGCGCCGCGGCTGGACCGCCACTACATTTCGGGGACT
GCGGGGGGGACGAGCCCCACCCGTAGAGCCCCAACACCGGGCGACGGCAGGCC
CCGTAGGGCTAGCCGCGCTCGAGGGAAGAAATGACGCTCGGACAGGCATGCCCG
GCAGATTGCTGCCGGGCGCAATGTGCGTTCAAAGATTCGATGATTCACTGAATTC
TGCAATTCACATTACTTATCGCATTTCGCTGCGTTCTTCATCGATGCCAGAACCAA
GAGATCCGTTGTTAAAAGTTTTGACTTGTTTTTTGTTTTGAATCAGAACGTGCAGT
ACGCTTTTTCAAATTTAGAGTTTGGCGCGGCCGGCGGGGACGGGGACGGGGA

Supplementary file 2: SSU and LSU sequences of the 5 most potent fungal isolates used for Blast search
LSU SEQUENCES:

Isolate 12: Galactomyces candidum 1

TGCCTTAGTAACGGCGAGTGAAGCGGCAAAAGCTCAAATTTGAAATCGGCCACC
AGGTCGAGTTGTAATTTGTAGATTGTATCTTGAGAGCGGATTAAAGTCTGTTGGA
ACACAGCGCCTTAGAGGGTGACAGCCCCGTAAAATCTATTCTCATTGTAAGATAC
TTTCGAAGAGTCGAGTTGTTTGGGAATGCAGCTCTAAGTGGGAGGTAAATTCCTT
CTAAAGCTAAATATTGACGAGAGACCGATAGCGAACAAGTACTGTGAAGGAAAG
ATGAAAAGCACTTTGAAAAGAGAGTGAAAAAGTACGTGAAATTGTTAAAAGGGA
AGGGTATTGAATCAGACTTGGTGCTGTTGTTCAACTGTGTTTTGGCACAGTGTAC
TCAGCAGTACTAGGCCAAGGTGGGGTGTTTGGGAGTGAAAAAGAAGTTGGAACG
TAACTCTTCGGAGTGTTATAGCCTACTTTCATAGCTCCTCAGGCGCCTCAGGACT
GCGCTTCGGCAAGGACCTTGGCATAATGATTCTATACCGCCCGTCTTGAAACACG
GACCAAGGAGTCTAACGTCTATGCGAGTGTTTGGGTGTAAAACCCGTACGCGTA
ATGAAAGTGAACGTAGATAGGAGCAGTAATGCGCACTATCGACCGATCCTGATG
TTTTCAGATGGATTTGAGTAAGAGCATAGCTGTTGGGACCCGAAAGATGGTGAA
CTATGCCTGAATAGGGTGAAGCCAGAGGAAACTCTGGTGGAGGCTCGTAGCGGT
TCTGACGTGCAAATCGATCGTCGAATTTGGGTATAGGGGCGAAAGACTAATCGA
ACCATCTAGTAGCTGGTTCCTGCCGAAGTTTCCCTCAGGATAGCAGAAGCTCGTA
TCAGTTTTATGAGGTAAAGCGAATGATTAGAGGTACTGGGGTCTATGTGACCTTA
ACC

Isolate 13: Galactomyces candidum 2

TGCCTTAGTAACGGCGAGTGAAGCGGCAAAAGCTCAAATTTGAAATCGGCCACC
AGGTCGAGTTGTAATTTGTAGATTGTATCTTGAGAGCGGATTAAAGTCTGTTGGA
ACACAGCGCCTTAGAGGGTGACAGCCCCGTAAAATCTATTCTCATTGTAAGATAC
TTTCGAAGAGTCGAGTTGTTTGGGAATGCAGCTCTAAGTGGGAGGTAAATTCCTT
CTAAAGCTAAATATTGACGAGAGACCGATAGCGAACAAGTACTGTGAAGGAAAG
ATGAAAAGCACTTTGAAAAGAGAGTGAAAAAGTACGTGAAATTGTTAAAAGGGA
AGGGTATTGAATCAGACTTGGTGCTGTTGTTCAACTGTGTTTCGGCACAGTGTAC
TCAGCAGTACTAGGCCAAGGTGGGGTGTTTGGGAGTGAAAAAGAAGTTGGAACG
TAACTCTTCGGAGTGTTATAGCCTACTTTCATAGCTCCTCAGGCGCCTCAGGACT
GCGCTTCGGCAAGGACCTTGGCATAATGATTCTATACCGCCCGTCTTGAAACACG
GACCAAGGAGTCTAACGTCTATGCGAGTGTTTGGGTGTAAAACCCGTACGCGTA
ATGAAAGTGAACGTAGATAGGAGCAGTAATGCGCACTATCGACCGATCCTGATG
TTTTCAGATGGATTTGAGTAAGAGCATAGCTGTTGGGACCCGAAAGATGGTGAA
CTATGCCTGAATAGGGTGAAGCCAGAGGAAACTCTGGTGGAGGCTCGTAGCGGT
TCTGACGTGCAAATCGATCGTCGAATTTGGGTATAGGGGCGAAAGACTAATCGA
ACCATCTAGTAGCTGGTTCCTGCCGAAGTTTCCCTCAGGATAGCAGAAGCTCGTA
TCAGTTTTATGAGGTAAAGCGAATGATTAGAGGT

Isolate 14: Isaria fumosorosea

GTAACGGCGAGTGAAGCGGCAACAGCTCAAATTTGAAATCTGGCCCCCGGTCC
GAGTTGTAATTTGCAGAGGATGCTTCGGGCGAGGTGCCTTCCGAGTTCCCTGGAA
CGGGACGCCACAGAGGGTGAGAGCCCCGTCTGGTCGGACACCGAGCCCGTGTGA
AGCTCCTTCGAAGAGTCGAGTAGTTTGGGAATGCTGCTCAAAACGGGAGGTATA
TGTCTTCTAAAGCTAAATATTGGCCAGAGACCGATAGCGCACAAGTAGAGTGAT
CGAAAGATGAAAAGCACTTTGAAAAGAGGGTTAAAAAGTACGTGAAATTGTTGA
AAGGGAAGCGCCCATGACCAGACTTGGGCCCGGTGAATCACCCGGCGTTCTCGC
CGGTGCACTTTGCCGGGCACAGGCCAGCATCAGTTTGGCGCGGGGGAGAAAGGC
TTCGGGAACGTGGCTCCCTCGGGAGTGTTATAGCCCGCTGCGCAATACCCTGCGC
CGGACTGAGGTACGCGCATCGCAAGGATGCTGGCGTAATGGTCATCAGCGACCC
GTCTTGAAACACGGACCAAGGAGTCGTCTTCGTATGCGAGTGTTCGGGTGTCAAA
CCCCTACGCGGAATGAAAGTGAACGCAGGTGAGAGCTTCGGCGCATCATCGACC
GATCCTGATGTTCTCGGATGGATTTGAGTAAGAGCATACGGGGCCGGACCCGAA
AGAAGGTGAACTATGCCTGTATAGGGTGAAGCCAGAGGAAACTCTGGTGGAAGC
TCGCAGCGGTTCTGACGTGCGAATCGATCGTCAAATATGGGCATGGGGGCGAAA
GACTAATCGAACCTTCTAGTAGCTGGTTTCCGCCGAAGTTTCCCTCAGGATAGCA
GTGTTGGACTCAGTTTTATGAGGTAAAGCGAATGATTAGGGACTCGGGGG

Isolate 17: Mucor hiemalis

TGATTTCCCTAGTAACGGCGAGTGAAGAGGAAAGAGCTCAAAGTTGGAACCTGT
TTGGCTTAGCTAAACCGGATTGTAAACTGTAGAAGTGTTTTCCAGACACGCCTGG
TAAAAAAGTCCTTTGGAACAGGGCATCATAGAGGGTGAGAATCCCGTCATTGGC
CAGAGCTGTTGTCTTTTGTGATACATTTTCAAAGAGTCAGGTTGTTTGGGAATGC
AGCCTAAATTGGGTGGTAAATCTCACCTAAAGCTAAATATTTGCGAGAGACCGA
TAGCGAACAAGTACCGTGAGGGAAAGATGAAAAGAACTTTGAAAAGAGAGTTA
AACAGTATGTGAAATTGTTAAAAGGGAACCGTTTGGAGCCAGACTGGCTTAACT
GTAATCAATCTAGGCTTTGGCCTGGATGCACTTGCGGTTTATGCCGGCCAACGAC
AGTTTTGTTTGAGGGAAAAAATTACATTGAATGTGGCCCTTCGGGGTGTTATAGC
TTTGTAAAAAATACCTTGGACTGGACTGAGGAACGCAGTGAATGCCTTTAGGCA
AGATTGCTGGGTGCTTTCGCTAATAAATGCTAGAATTTCTGCTTCGGGTGGTGCT
AGTGTTTAAAGGAGGAACTCGCTTAGTATATTTTTTATTCGCTTAGGTTGTTGGCT
TAATGACTCTAAATGACCCGTCTTGAAACACGGACCAAGGAGTCCACCATAAGT
GCGAGTATTTGGGTGACAAACCCATATGCGCAAGGAAACTGATTGATACGAAGG
CTTTAAGCTGGCAGTATCACCCGGCGTTGACGTTTTATACTGAACTGACCGAGGT
AAAGCACTTATGATGGGACCCGAAAGATGGTGAACTATGCCTGAATAGGGTGAA
GCCGGAGGAAACTCCGGTGGAGGCTCGTAGCGATTCTGACGTGCAAATCGATCG
TCAAATTTGGGTATAGGGGCGAAAGACTAATCGAACCATCTAGTA

SSU SEQUENCES:

Isolate 12: Galactomyces candidum 1

GTGAAACTGCGAATGGCTCATTAAATCAGTTATCGTTTATTTGATATTACATTACT
ACTTGGATAACCGTGGTAATTCTAGAGCTAATACATGCTAAAACGGCCGGGTTCC
CGGCTGGTATTTATTAGATAAAAAACCAATGCCTTCGGGCTCTATGGTGAATCAT
AATAACTTGTCGAATCGCATGGCCTTGTGCTGGCGATGGTTCATTCAAATTTCTG
CCCTATCAACTTTCGATGGTAGGATAGAGGCCTACCATGGTTTTAACGGGTAACG
GGGAATCAGGGTTCGATTCCGGAGAGGGAGCCTGAGAAACGGCTACCACATCCA
AGGAAGGCAGCAGGCGCGCAAATTACCCAATCCTGACACAGGGAGGTAGTGAC
AATAAATAACGATACGGGGCCTATTAGGTCTCGTAATTGGAATGAGAACAATTT
AAATACCTTAACGAGGAACAATTAGAGGGCAAGTCTGGTGCCAGCAGCCGCGGT
AATTCCAGCTCTGATAGTATATATTAAAGTTGTTGCAGTTAAAAAGCTCGTAGTT
GAAACTTGGGTGTGTAGGGGCGGTCTCTTTTAGAGTACTACCCTGAAACATCTTT
CTTTGGTGTAAACTCTTTATTCACTTAAGGAGTGTAAACCAAACATTTACTTTGAA
AAAATTAGAGTGTTCAAAGCAGGCCTTTGCTCGAATATATTAGCATGGAATAATA
GAATAGGACGTATGGTTCTATTTTGTTGGTTTCTAGGACCGTCGTAATGATTAAT
AGGGACGGTCGGGGGCATCAGTATTCAGTTGTCAGAGGTGAAATTCTTGGATTTA
CTGAAGACTAACTACTGCGAAAGCATTTGCCAAGGACGTTTTCATTAATCAAGAA
CGAAAGTTAGGGGATCGAAGACGATCAGATACCGTCGTAGTCTTAACCGTAAAC
TATGCCGACTAGGGATCGGAGGGCGTTATAATAACCTCTCCGGCA

Isolate 13: Galactomyces candidum 2

TATACAGTGAAACTGCGAATGGCTCATTAAATCAGTTATCGTTTATTTGATATTA
CATTACTACTTGGATAACCGTGGTAATTCTAGAGCTAATACATGCTAAAACGGCC
GGGTTCCCGGCTGGTATTTATTAGATAAAAAACCAATGCCTTCGGGCTCTATGGT
GAATCATAATAACTTGTCGAATCGCATGGCCTTGTGCTGGCGATGGTTCATTCAA
ATTTCTGCCCTATCAACTTTCGATGGTAGGATAGAGGCCTACCATGGTTTTAACG
GGTAACGGGGAATCAGGGTTCGATTCCGGAGAGGGAGCCTGAGAAACGGCTACC
ACATCCAAGGAAGGCAGCAGGCGCGCAAATTACCCAATCCTGACACAGGGAGGT
AGTGACAATAAATAACGATACGGGGCCTATTAGGTCTCGTAATTGGAATGAGAA
CAATTTAAATACCTTAACGAGGAACAATTAGAGGGCAAGTCTGGTGCCAGCAGC
CGCGGTAATTCCAGCTCTGATAGTATATATTAAAGTTGTTGCAGTTAAAAAGCTC
GTAGTTGAAACTTGGGTGCGTAGGGGCGGTCTCTTTTAGAGTACTACCCTGAAAC
ATCTTTCTTTGGTGTAAACTTTCTATTTATTTAGGAAGTGTAAACCAAACATTTAC
TTTGAAAAAATTAGAGTGTTCAAAGCAGGCCTTTGCTCGAATATATTAGCATGGA
ATAATAGAATAGGACGTATGGTTCTATTTTGTTGGTTTCTAGGACCGTCGTAATG
ATTAATAGGGACGGTCGGGGGCATCAGTATTCAGTTGTCAGAGGTGAAATTCTTG
GATTTACTGAAGACTAACTACTGCGAAAGCATTTGCCAAGGACGTTTTCATTAAT
CAAGAACGAAAGTTAGGGGATCGAAGACGATCAGATACCGTCGTAGTCTTAACC
GTAAACTATGCCGACTAGGGATCGGAGGG

Isolate 14: Isaria fumosorosea

GCATTATACAGCGAAACTGCGAATGGCTCATTATATAAGTTATCGTTTATTTGAT
AGTACCTTACTACTTGGATAACCGTGGTAATTCTAGAGCTAATACATGCTAAAAA
TCCCGACTTCGGAAGGGATGTATTTATTAGATTAAAAACCAATGCCCTCTGGGCT
CCTTGGTGATTCATGATAACTCTTCGAATCGCACGGCCTTGCGCCGGCGATGGTT
CATTCAAATTTCTTCCCTATCAACTTTCGATGTTTGGGTATTGGCCAAACATGGTT
GCAACGGGTAACGGAGGGTTAGGGCTCGACCCCGGAGAAGGAGCCTGAGAAAC
GGCTACTACATCCAAGGAAGGCAGCAGGCGCGCAAATTACCCAATCCCGATTCG
GGGAGGTAGTGACAATAAATACTGATACAGGGCTCTTTTGGGTCTTGTAATTGGA
ATGAGTACAATTTAAATCCCTTAACGAGGAACAATTGGAGGGCAAGTCTGGTGC
CAGCAGCCGCGGTAATTCCAGCTCCAATAGCGTATATTAAAGTTGTTGTGGTTAA
AAAGCTCGTAGTTGAACCTTGGGCCTGGCTGGCCGGTCCGCCTCACCGCGTGCAC
TGGTCCGGCCGGGCCTTTCCCTCTGTGGAACCCCATGCCCTTCACTGGGCGTGGC
GGGGAAACAGGACGTTTACTTTGAAAAAATTAGAGTGCTCCAGGCAGGCCTATG
CTCGAATACATTAGCATGGAATAATGAAATAGGACGCGTGGTTCTATTTTGTTGG
TTTCTAGGACCGCCGTAATGATTAATAGGGACAGTCGGGGGCATCAGTATTCAAT
TGTCAGAGGTGAAATTCTTGGATTTATTGAAGACTAACTACTGCGAAAGCATTTG
CCAAGGATGTTTTCATTAATCAGGAACGAAAGTTAGGGGATCGAAGACGATCAG
ATACCGTCGTAGTCTTAACCATAAACTATGCCGACTAGGGATCGGACGATGTTAT
TTTTT

Isolate 17: Mucor hiemalis

AATAAATTTATATTGTGAAACTGCGAATGGCTCATTAAATCAGTTATGATCTACG
TGACATATTCTTTACTACTTGGATAACCGTGGTAATTCTAGAGCTAATACATGCA
AAAAAACCCTGACTTCGGAAGGGGTGCACTTATTAGATAAAGCCAACGCGGGGT
AAAACCTGTTTCCCTTGGTGATTCATAATAATTAAGCGGATCGCATGGCCTTGTG
CTAGCGACGGTCCACTCGATTTTCTGCCCTATCATGGTTGAGATTGTAAGATAGA
GGCTTACAATGCCTACAACGGGTAACGGGGAATTAGGGTTCGATTCCGGAGAGG
GAGCCTGAGAAACGGCTACCACATCCAAGGAAGGCAGCAGGCGCGCAAATTACC
CAATCCCGACACGGGGAGGTAGTGACAATAAATAACAATGCAGGGCCTTTAAGG
TCTTGCAATTGGAATGAGTACAATTTAAATCCCTTAACGAGGATCAATTGGAGGG
CAAGTCTGGTGCCAGCAGCCGCGGTAATTCCAGCTCCAATAGCGTATATTAAAGT
TGTTGCAGTTAAAACGTCCGTAGTCAAATTTTAGTCTTTAGGCGAAGTGGCCTGG
TCTTCATTGATCAAGCTCGTTTCTGCCGAGACTTTTTTTTTGGTTATGCTACTGTTG
GCTTCGGTCGGCGGTAGTCTCTAGCCAAATGATTACCATGAGCAAATCAGAGTGT
TTAAAGCAGGCTTTCAAGCTTGAATGTGTTAGCATGGAATAATGAAATATGACTT
TAGTCCCTATTTCGTTGGTTCAGGAACTTAAGTAATGATGAATAGAAACGGTTGG
GGGCATTTGTATTTGGTCGCTAGAGGTGAAATTCTTGGATTGACCGAAGACAAAC
TACTGCGAAAGCATTTGACCCAGGACGTTTTCATTGATCAAGGTCTAAAGTTAAG
GGATCGAAGACGATTAGATACCGTCGTAGTCTTAACCACAAACTATGCCGACTA
GCGATTGGG

Supplementary file 3: ITS-based neighbor-joining-based phylogenetic trees of the 22 fungal isolates

<u>Isolate 1</u>

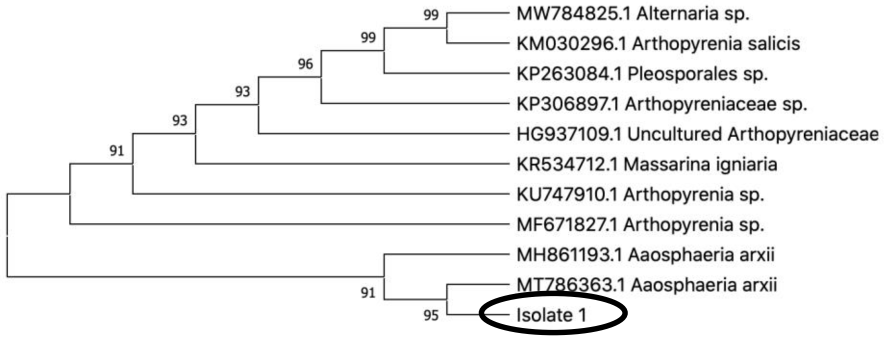

<u>Isolate 2</u>

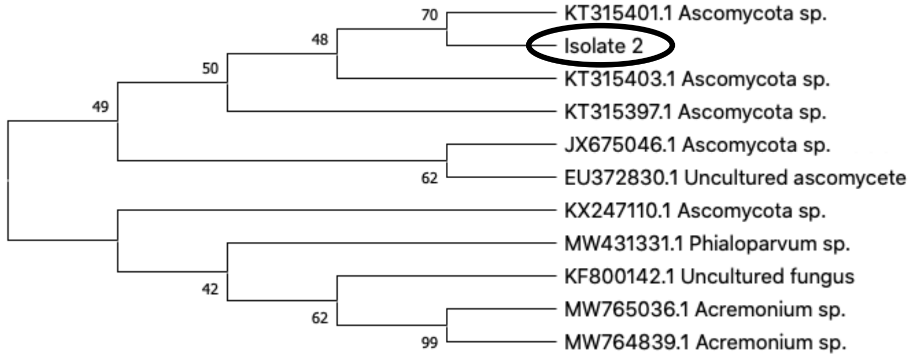

<u>Isolate 3</u>

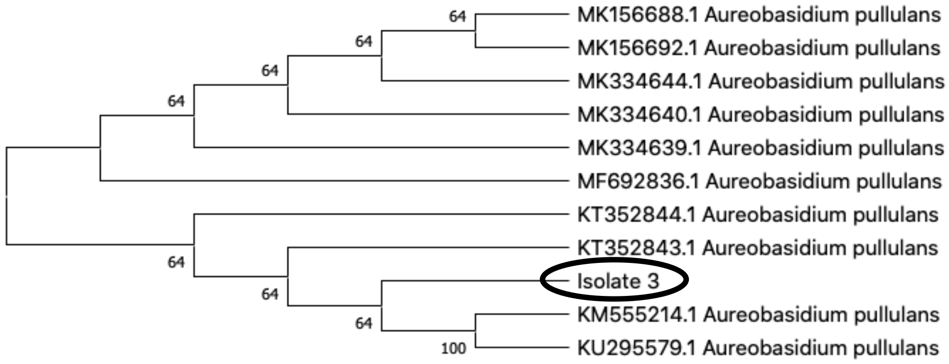

<u>Isolate 4</u>

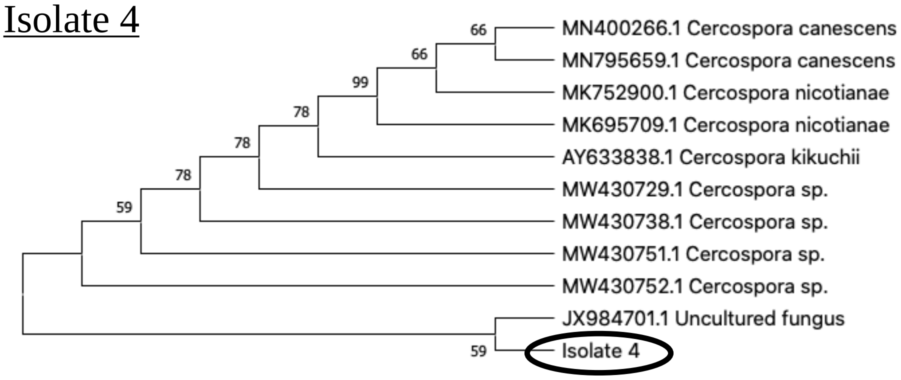

Isolate 5

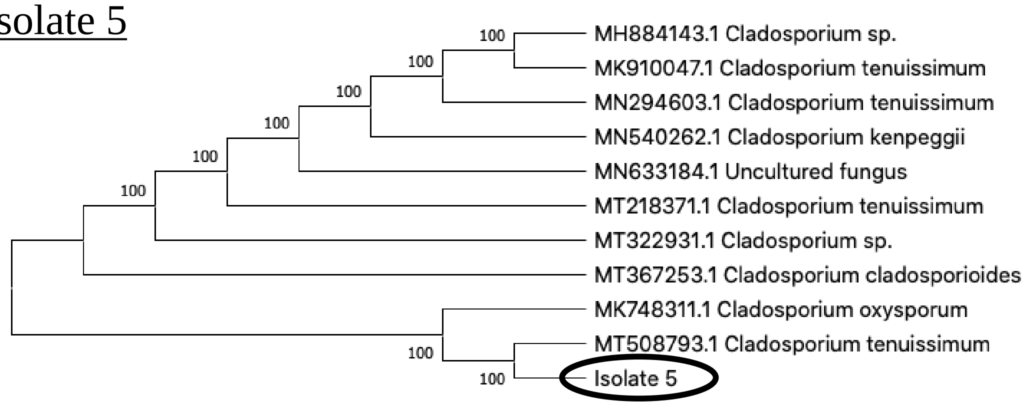

Isolate 6

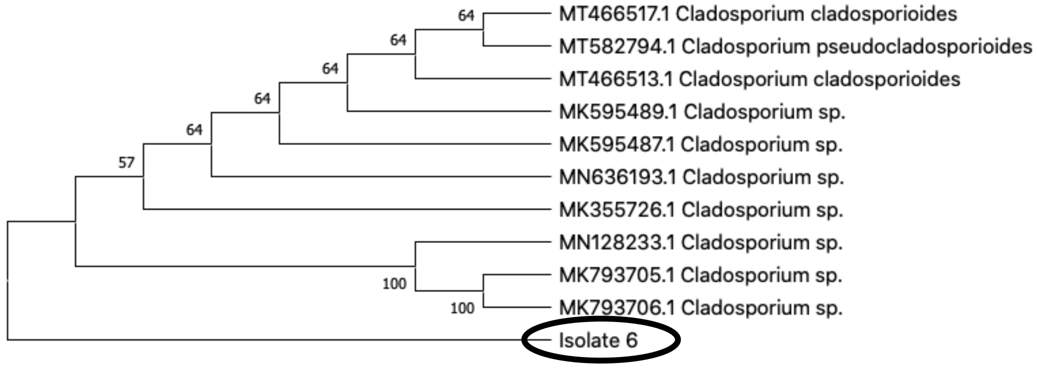

Isolate 7

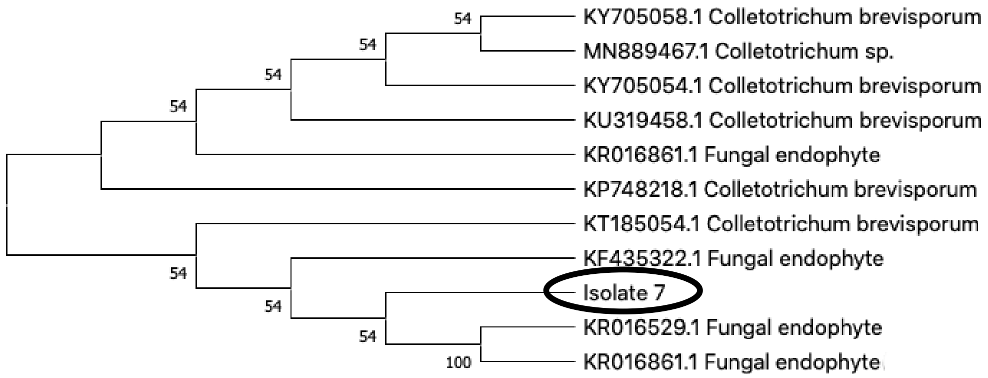

Isolate 8

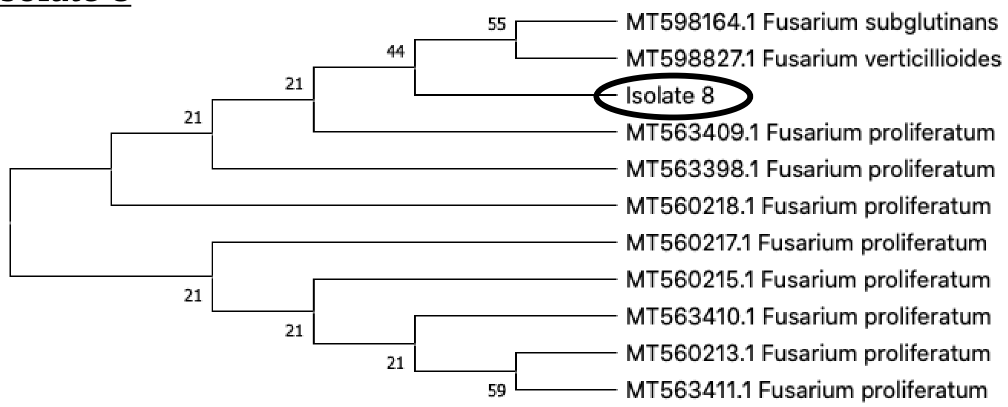

Isolate 9

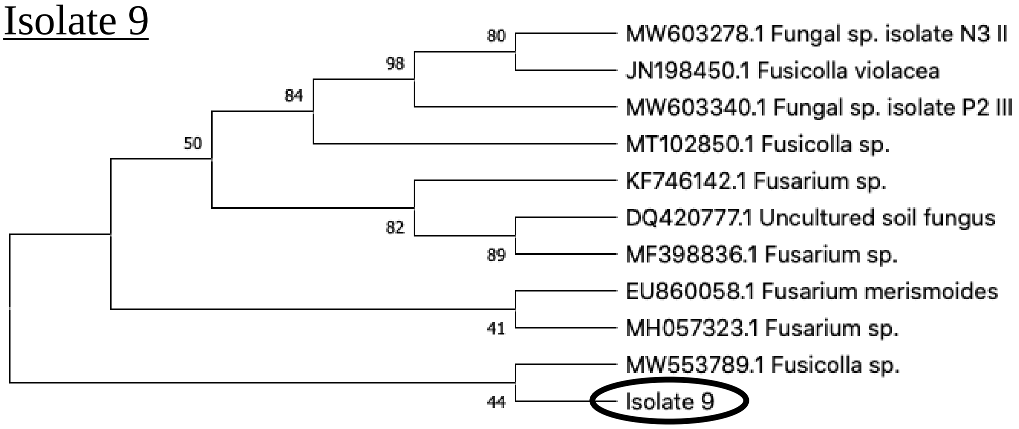

Isolate 10

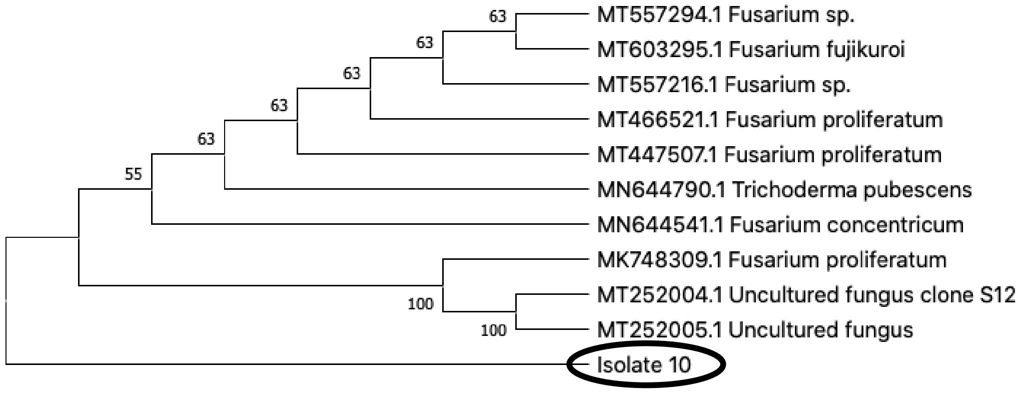

Isolate 11

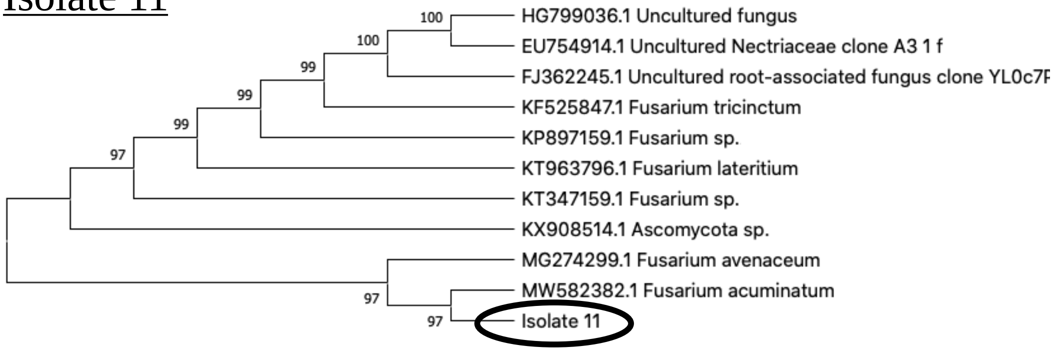

Isolate 12

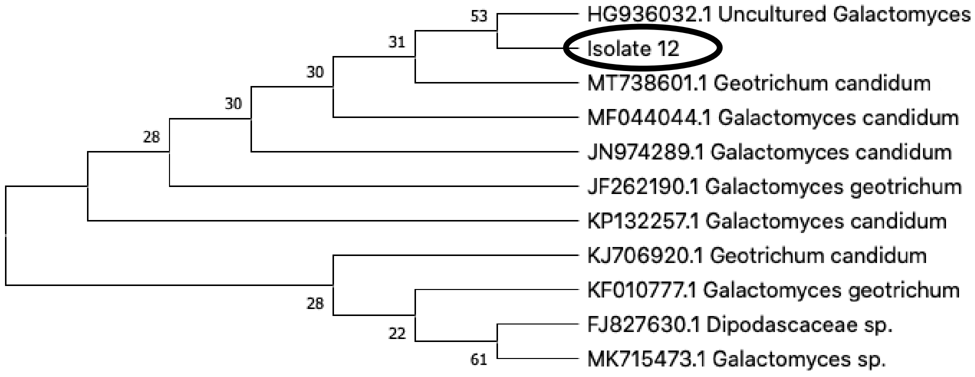

Isolate 13

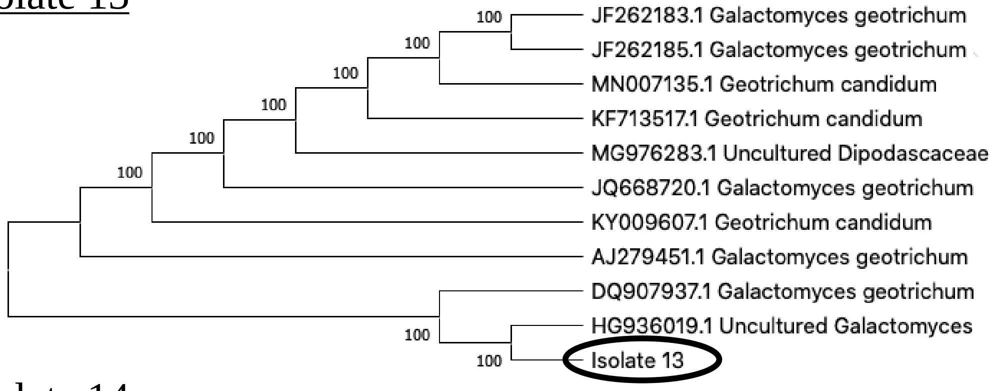

Isolate 14

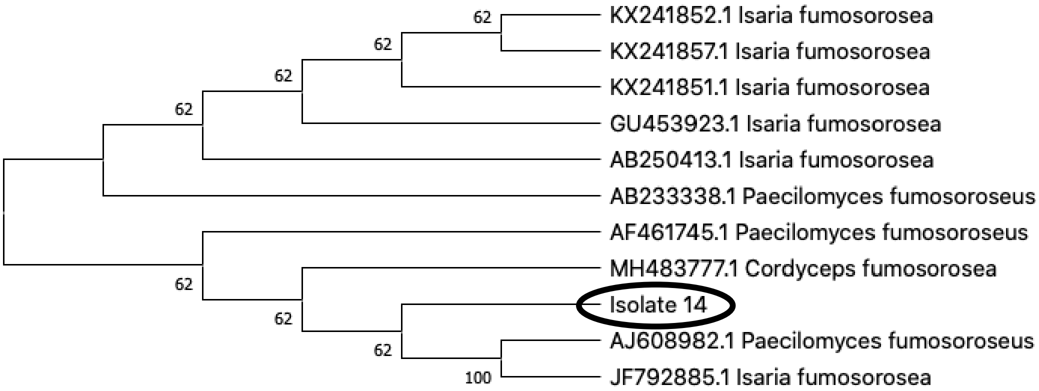

Isolate 15

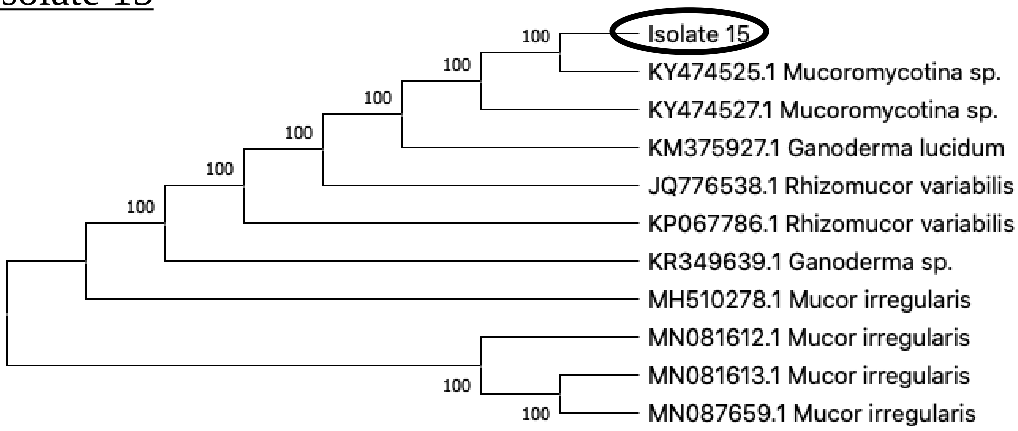

Isolate 16

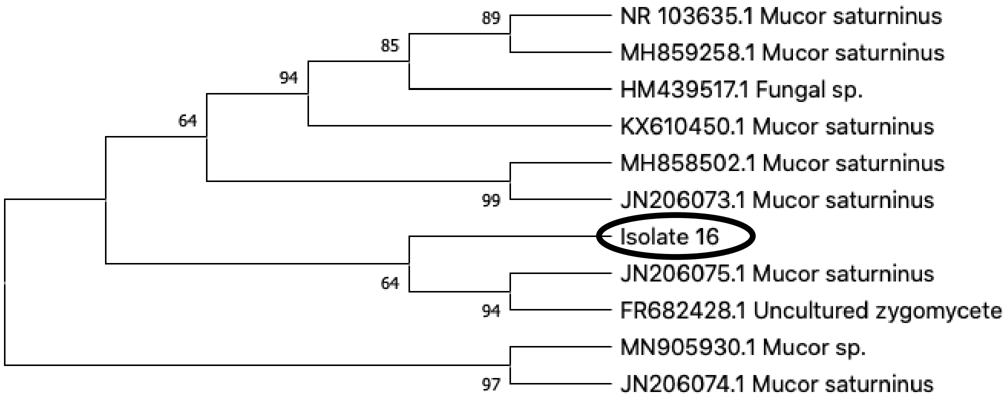

Isolate 17

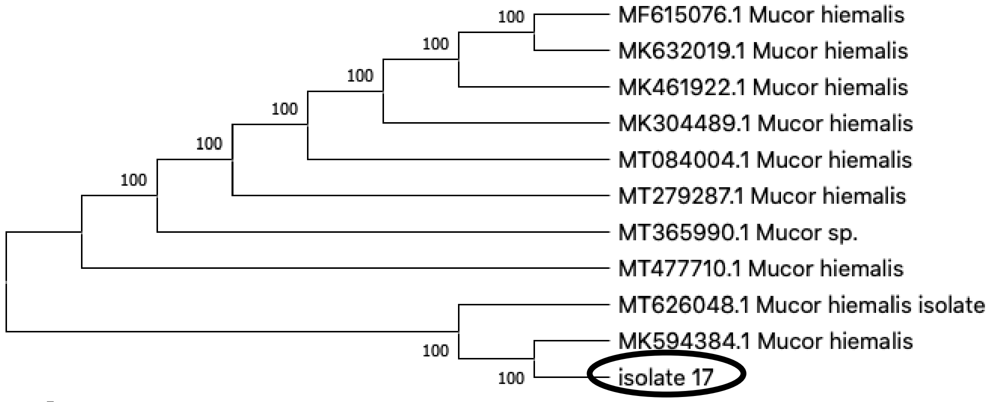

Isolate 18

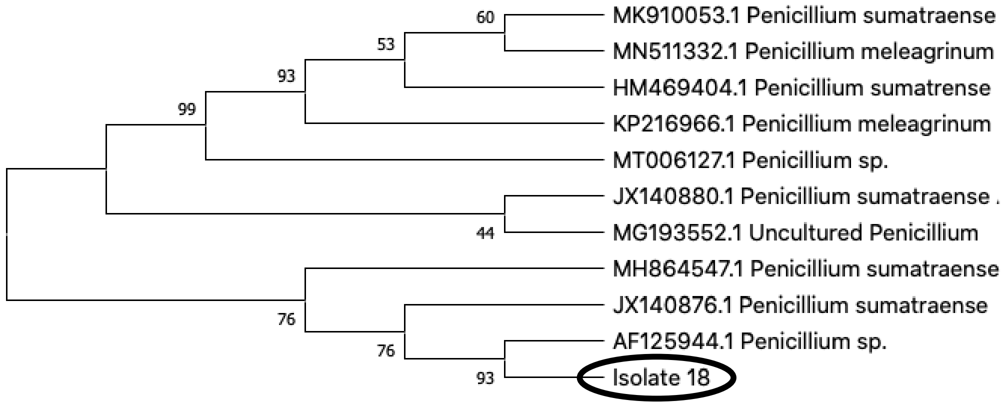

Isolate 19

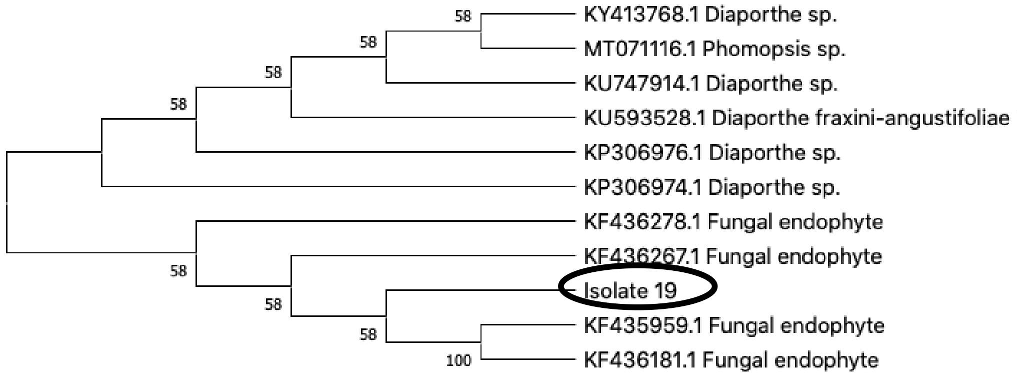

Isolate 20

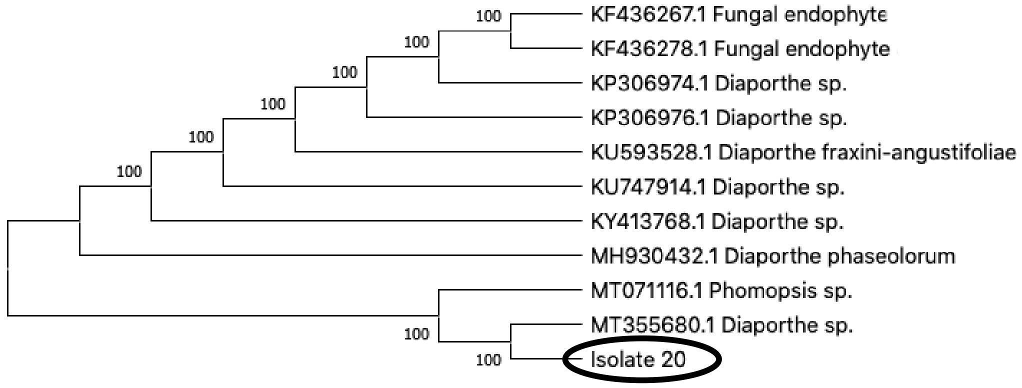

<u>Isolate 21</u>

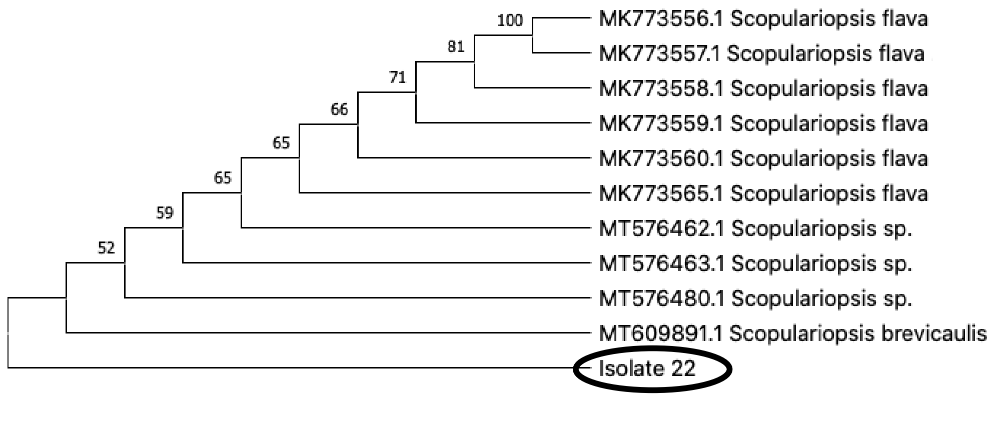

<u>Isolate 22</u>

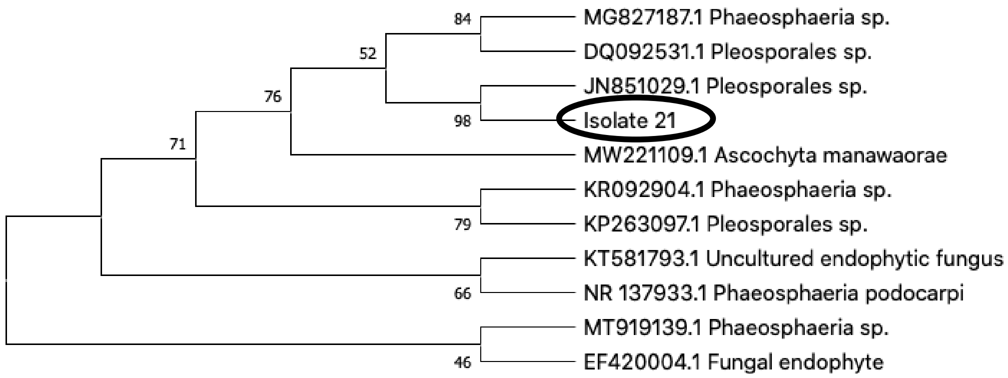

Supplement: Supplementary file 1 [file Data_Sheet_1.pdf]
